# Genome-Informed Loop-Mediated Isothermal Amplification (LAMP) Assays for Detection and Differentiation of *Ralstonia pseudosolanacearum* Phylotypes I and III

**DOI:** 10.3390/ijms27208976

**Published:** 2026-10-10

**Authors:** Brijesh B. Karakkat, Qi Huang, Schyler O. Nunziata, Jarred Yasuhara-Bell

**Affiliations:** 1 Department of Entomology and Plant Pathology, North Carolina State University, Raleigh, NC 27607, USA; brijesh.karakkat@usda.gov; 2 Plant Pathogen Confirmatory Diagnostics Laboratory (PPCDL), Science and Technology (S&T), Plant Protection and Quarantine (PPQ), Animal and Plant Health Inspection Service (APHIS), United States Department of Agriculture (USDA), Laurel, MD 20708, USA; schyler.o.nunziata@usda.gov; 3 Floral and Nursery Plants Research Unit, United States National Arboretum, USDA Agricultural Research Service (ARS), Beltsville, MD 20705, USA; qi.huang@usda.gov

**Keywords:** *Ralstonia pseudosolanacearum*, phylotype I, phylotype III, bacterial wilt, detection, differentiation, loop-mediated amplification, LAMP

## Abstract

The *Ralstonia solanacearum* species complex (RSSC) causes bacterial wilt in a broad range of economically important hosts. The RSSC has been reclassified into three species, of which *R. solanacearum* sequevars 1 and 2 in phylotype IIB are designated as Select Agents in the U.S. Many rapid tests for “*R. solanacearum*” target the RSSC and thus cross-react with *R. pseudosolanacearum*, which is endemic to the U.S. To avoid false positive identifications and unnecessary triggering of Select Agent measures, four portable LAMP-based screening assay variations were developed and validated for specific detection of *R. pseudosolanacearum*: real-time LAMP assays for detection of *R. pseudosolanacearum* (1) and differentiation of *R. pseudosolanacearum* phylotypes I and III in the AmpliFire^®^ Isothermal Fluorometer (2) or Genie^®^ III (3), and a colorimetric LAMP for detection of *R. pseudosolanacearum* (4). All assays demonstrated high sensitivity (10–50 colony-forming units (CFU)/reaction) and specificity; 100% analytical specificity and selectivity based on 177 RSSC isolates (81 phylotype I, 70 phylotype II, 15 phylotype III and 11 phylotype IV) and 53 non-RSSC bacterial pathogens on relevant hosts and soil, and 100% diagnostic specificity and sensitivity based on testing inoculated plants and a Euphresco sample panel. The assays presented here represent useful diagnostic tools that can be deployed on-site to aid domestic survey and detection efforts.

## 1. Introduction

The *Ralstonia solanacearum* species complex (RSSC) causes bacterial wilt in a broad range of hosts. This group has been confounded by various naming schemes. Race and biovar were terms used to differentiate lineages previously. The RSSC was later reclassified into four major phylotypes based on phylogenetic analyses of the 16S–23S internal transcribed spacer (ITS) region. These phylotypes are associated with geographic origin; Phylotype I originated from Asia, II from the Americas, III from Africa and the Indian Ocean, and IV from Australia, Indonesia, and Japan [1]. More recently, these phylotypes were grouped into three species: *R. pseudosolanacearum* (phylotypes I and III), *R. solanacearum* (phylotype II), and *R. syzygii* (phylotype IV) [2,3].

*Ralstonia solanacearum* (phylotype IIB) sequevars 1 and 2 (based on the sequence of the endoglucanase (*egl*) gene) were historically known as Race 3 biovar 2 (R3b2). These strains are quarantine pests in Europe and Canada and are a strictly regulated Select Agent pathogen in the U.S. The ImmunoStrip^®^ test for *R. solanacearum* (Agdia Inc.) is a quick and easy field-ready test; however, this test detects the entire RSSC. Thus, it can cross-react with *R. pseudosolanacearum* (phylotype I), which is endemic in the U.S. *Ralstonia pseudosolanacearum* is the most prevalent cause of bacterial epidemics in tropical and subtropical environments and is sometimes associated with severe outbreaks in temperate regions [1,4]. Novel phylotype I strains of *R. pseudosolanacearum* have appeared in the U.S. and Europe in the last decade [4,5,6], and have continued to spread throughout the world [7,8,9,10,11,12,13,14,15,16,17]. While phylotype I strains of *R. pseudosolanacearum* have been reported in the U.S., phylotype III strains remain exotic. Having a field-deployable test that can be used instead of the ImmunoStrip^®^ and specifically detect domestic *R. pseudosolanacearum*, ruling out possible *R. solanacearum* phylotype IIB sequevars 1 and 2 infection, would avoid placing Select Agent status on samples unnecessarily. Additionally, such an assay could be deployed to detect potentially exotic *R. pseudosolanacearum* in imported plant material, e.g., at PPQ Plant Inspection Stations.

Isothermal nucleic acid amplification technologies obviate the need for a thermocycler by allowing amplification to occur at a single temperature. This property enables reactions to be run in simplified devices that can hold a specified constant temperature, allowing portability, which is a key element for field diagnostics. Additionally, several properties of the enzymes used for isothermal amplification, which differ from those of PCR, afford them varying degrees of tolerance to inhibitors. Tolerance to inhibitors allows compatibility with simplified and crude nucleic acid extraction procedures. Several isothermal nucleic acid amplification technologies are available, including recombinase polymerase amplification (RPA) [18] and loop-mediated isothermal amplification (LAMP) [19,20,21].

Among the isothermal nucleic acid methods currently available, LAMP is the most widely researched. There are multiple primer design tools available, as well as multiple companies producing a variety of LAMP reagents for various applications. In addition, LAMP results can be assessed in various formats, including lateral flow, and both endpoint and real-time fluorescence using dyes or probes; RPA assays are also available in these formats. Colorimetric LAMP assays are also available, and results can be viewed at end-point as a simple color change, using dyes that respond to double-stranded DNA [22,23,24], or based on changes in pH [25] or concentration of metal ions such as Mg^2+^ [26,27]. Colorimetric assays do not require high-tech equipment to read fluorescence data, thus can be run in a simple device capable of holding the desired running temperature.

Several LAMP-based [28,29,30,31,32,33,34,35,36] and RPA-based [37,38] assays have been reported in the literature over the years; however, most of these assays target the entire RSSC or very specific strains, such as phylotype IIB sequevars 1 and 2 (R3b2) or host-specific strains. Only a single publication was found describing a LAMP assay designed to detect all *R. pseudosolanacearum* phylotype I strains [36]; no assays were identified in the literature for detection of *R. pseudosolanacearum* phylotype III strains. Phylotype III strains are endemic to Africa, but their global relevance has not been evaluated. Increased global trade and travel have allowed the spread of many pathogens to new areas. The potential impacts of these strains on foreign agriculture are unknown. Movement of pathogens to new areas can create new disease dynamics due to different climates, different ecosystems, presence of different host species and/or different varieties of the same host species. Thus, the importance of *R. pseudosolanacearum* phylotype III strains cannot be ignored. The aim of this study was to develop and validate a LAMP assay that can detect all *R. pseudosolanacearum* strains (phylotypes I and III), as well as differentiate between phylotypes I and III, to aid in surveillance and detection efforts to safeguard U.S. agriculture and facilitate safe trade.

## 2. Results

### 2.1. Target Identification

Comparative genomic analysis identified 151 genes as being unique to all *R. pseudosolanacearum* (phylotypes I and III), while 844 and 886 genes were identified as being unique to phylotypes I and III, respectively. Results were analyzed manually to look for genes > 450 bp to increase the probability for primer design. Additionally, potential target genes representing mobile genetic elements (e.g., transposons, phages, etc.) were excluded. Initially, a target specific to all *R. pseudosolanacearum* (phylotypes I and III) was sought for assay development. Results from BLAST analysis against a *Ralstonia* genome database revealed several candidates. Results were filtered manually to select targets that matched all known and available *R. pseudosolanacearum* genomes with >90% sequence identity and <80% identity with other organisms. Several candidates were identified, and primer design was attempted. While the candidate genes were present in all *R. pseudosolanacearum*, sequence differences between phylotypes I and III made it impossible to properly design LAMP primers.

A new strategy was developed to use markers specific for phylotypes I and III and mix them in a “duplex” assay that would allow detection of all *R. pseudosolanacearum* and/or differentiation of phylotypes I and III. The same process was followed to identify candidate targets specific to phylotypes I and III. Several candidate targets were identified that met all filtering criteria. Attempts were made to design primers to all identified potential targets, but primer sets to only one phylotype I-specific and four phylotype III-specific targets were designed successfully. For phylotype I, a conserved protein of unknown function (MICFAM_ID 637794 [39,40,41,42,43,44], designated as CP) was identified as a useful diagnostic marker, matching only to *R. pseudosolanacearum* phylotype I strains and allowing primer design (locus tag RSc1496 in GMI1000). Preliminary tests with select strains revealed CP to be a promising target for phylotype I diagnostics.

For phylotype III, a RICIN domain-containing protein (singleton; does not have MICFAM_ID), a hypothetical protein (MICFAM_ID 81102), another hypothetical protein (singleton), and a type II secretion system F family protein (MICFAM_ID 81109), were identified as useful diagnostic markers, matching only to *R. pseudosolanacearum* phylotype III strains and allowing primer design (locus tags PG907_RS04575, PG907_RS00100, PG907_RS05085 and PG907_RS00135 in RUN2340, respectively). Sequences were input into Geneious Prime^®^ 2023.0.4, and all target amplicons were extracted and rescreened using NCBI BLASTn against multiple databases. Results suggested that amplicons were specific to their respective phylotypes. After preliminary testing of the primers with select strains, the hypothetical protein (MICFAM_ID 81102, locus tag PG907_RS00100, designated as HP) showed promise for phylotype III diagnostics and was evaluated further in this study.

### 2.2. Assay Sensitivity

The limit of detection (LoD) was determined using serial dilutions of *R. pseudosolanacearum* DNA. Dilutions were tested with the 16S PCR to establish relative C_t_ values and enable comparison of the LAMP assays to real-time PCR assay data. Results revealed similar C_t_ values across all three strains for corresponding dilutions (Supplementary Table S1). LAMP data revealed that the LoD for extracted DNA tested with Assays 1 and 2 in the QuantStudio™ 5 Real-Time PCR System and CFX Opus Real-Time PCR System, respectively, was strain-dependent: ~1300 genome equivalent copies (GEC)/reaction, ~800 GEC/reaction and ~80 GEC/reaction for phylotype I strain 202402-02, and phylotype II strains UW386 and UW472, respectively.

LoD determinations were repeated using heat-lysed cell suspensions in TE buffer to replicate field-applicable extractions; real-time LAMP assays were performed using appropriate portable instruments. The LoD for phylotype I strain 202402-02 and phylotype III strain UW685 cell suspensions was as low as ~10 CFU/reaction and ~20 CFU/reaction for all tests, respectively, except Assay 3 in the Genie^®^ III, which was ~20–40 CFU/reaction, based on 100% positivity from three replicate wells. The LoD for heat-lysed cell suspensions revealed an approximate 100-fold increase in sensitivity when compared to using purified DNA. Cell suspensions were tested with the 16S PCR, and C_t_ values were used to back-calculate concentration using a standard curve produced using dilution data: $Conc.=e^{\frac{C_{t}-36.501}{-1.718}}$. Calculated concentrations confirmed the expected CFU/reaction of the dilutions. The LoD for phylotype I strain 202402-02 and phylotype III strain UW685 cell suspensions was also evaluated for the colorimetric LAMP assay (Assay 4): ~10 CFU/reaction.

To determine the effect of host compounds on assay performance, cell suspensions in healthy host (tomato, geranium and oleander) exudates were used to simulate actual testing in the field. Test data revealed that the LoD of all assays remained unaffected by the presence of host compounds in the sample, except for geranium. The LoD for phylotype I strain 202402-02 and phylotype III strain UW685 cell suspensions in geranium exudate was 50–250 CFU/reaction and ~40 CFU/reaction, respectively. Cumulative results suggested that the sensitivity of real-time and colorimetric LAMP assays was sufficient for testing symptomatic plants.

### 2.3. Assay Specificity

LAMP specificity data were obtained for Assays 1 and 2 in the QuantStudio™ 5 Real-Time PCR System and CFX Opus Real-Time PCR System for all strains included in this study (Supplementary Table S2); the assays demonstrated 100% specificity. All *R. pseudosolanacearum* strains produced positive results for Assay 1 and Assay 2, and Assay 2 was able to differentiate phylotypes I and III appropriately with 100% inclusivity. Negative results were produced for all other organisms tested (100% exclusivity), as well as healthy plant tissue (100% selectivity), except for nine phylotype II strains. Eight strains produced late positive reactions (20–25 min) with the LAMP assays for phylotype I, suggesting low-level contamination by a phylotype I strain; the ninth strain (UW425) produced positive results at ~15 min, suggesting a higher level of contamination. DNA extracted from these nine strains was tested with the Fegan and Prior [1] and Paudel et al. [45] conventional PCR (cPCR) assays. These assays were performed using only the published phylotype I and II primer sets in simplex format. Results demonstrated that these strains were phylotype II, as seen by the bright bands for the phylotype II primer sets (Supplementary Figure S1). Weak bands for phylotype I were seen for all strains, suggesting contamination by a phylotype I strain (Supplementary Figure S1); UW425 had the brightest band, corroborating LAMP results. Additionally, RUN302, RUN141 (CFBP7027) and RUN160 (JT516) were tested with the Robène et al. [36] LAMP assay and produced positive amplification, suggesting the presence of phylotype I. PCR and LAMP results demonstrated true contamination by an unknown phylotype I in these samples, as four independent assays targeting different phylotype I-specific markers all produced positive results. These results confirmed the 100% specificity of the LAMP assays developed in this study.

**Figure 1:**
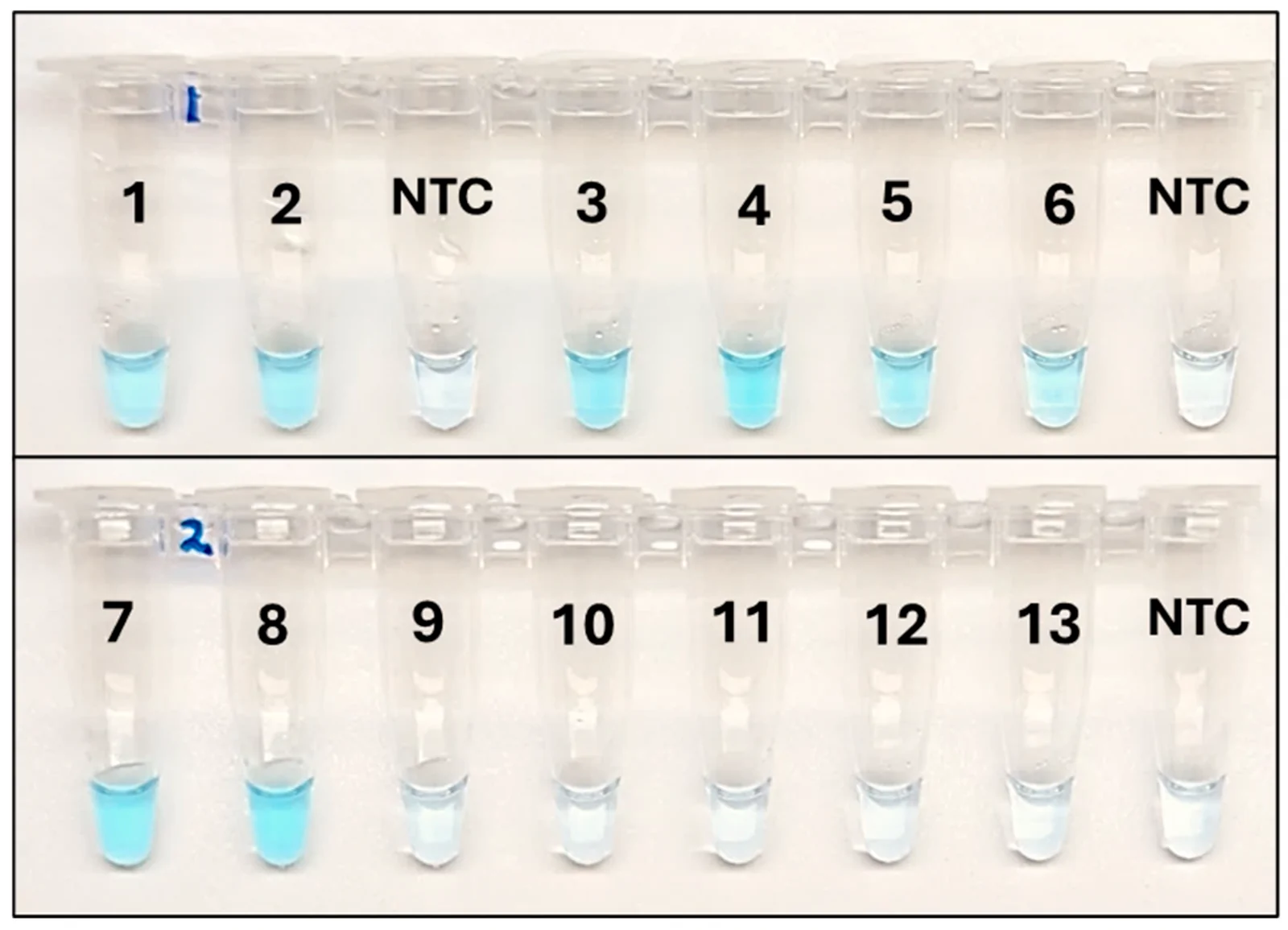
Small-scale specificity testing results for the colorimetric LAMP assay (Assay 4). Positive results are viewed as a color change from clear (color before incubation) to blue. (1) 202402-02 (I-30); (2) UW361 (I-12); (3) UW386 (III-23); (4) UW472 (III-43); (5) CFBP3059 (III-23); (6) J25 (III-20); (7) JT525 (III-19); (8) LMG 9673^T^ (III-19); (9) 202401-06 (IIC-7); (10) CFBP8695 (IIB-1); (11) MolK2 (IIB-3); (12) LMG 27703^T^ (IV-10); (13) LMG 10661^T^ (IV-9); NTC = no-template control. Only *Ralstonia pseudosolanacearum* (Phylotypes I and III) samples produced positive results with the colorimetric LAMP assay.

Due to the limited throughput of the portable instruments (eight-well capacity), specificity for all strains was initially checked with Assays 1 and 2 using real-time PCR equipment. A smaller-scale specificity test panel was used to corroborate specificity in the portable instruments; specificity was not expected to change as only the HP-probe fluorophore was changed to accommodate different instruments. Testing in portable instruments confirmed 100% specificity, detecting only *R. pseudosolanacearum* (Assay 1) and being able to differentiate phylotypes I and III (Assays 2 and 3) (Supplementary Table S3). The colorimetric assay (Assay 4) was also tested and revealed 100% specificity (Figure 1).

It is possible for assays to only show cross-reactivity to non-target organisms when they are present in high concentrations. To test this, *Ralstonia* strains 202402-02 (I-30), UW386 (III-23), 202401-06 (IIC-7) CFBP8695, (IIB-1), Molk2 (IIB-3), LMG27703^T^ (IV-10) and LMG10661^T^ (IV-9) were tested under high-titer (~500 ng/reaction) conditions in the AmpliFire^®^ Isothermal Fluorometer using Assays 1 and 2. Results showed that the assays remained specific for *R. pseudosolanacearum* phylotypes I and III, even with high DNA load from other closely related *Ralstonia* spp. Additionally, no adverse effects on positive amplification were observed with high DNA load.

### 2.4. Diagnostic Sensitivity and Specificity

Thirty-four diagnostic samples from the U.S. and Guam testing positive for *Ralstonia* spp. by 16S PCR previously were used to determine diagnostic specificity and sensitivity. Diagnostic specificity and sensitivity data were obtained for Assays 1 and 2 in the QuantStudio™ 5 Real-Time PCR System and CFX Opus Real-Time PCR (Supplementary Table S4). These assays demonstrated 100% diagnostic sensitivity, producing positive results for Assays 1 and 2 for all samples known to contain *R. pseudosolanacearum* (N = 22). Additionally, Assay 2 accurately determined that the *R. pseudosolanacearum* strains were phylotype I, as samples were phylotyped previously by *egl* (endoglucanase gene) sequencing; phylotype III has not been reported in the U.S. Negative results were produced for all samples known to contain *R. solanacearum* (N = 12) (100% diagnostic specificity). *Ralstonia syzygii* has not been reported in the U.S.; therefore, no diagnostic samples were available.

**Figure 2:**
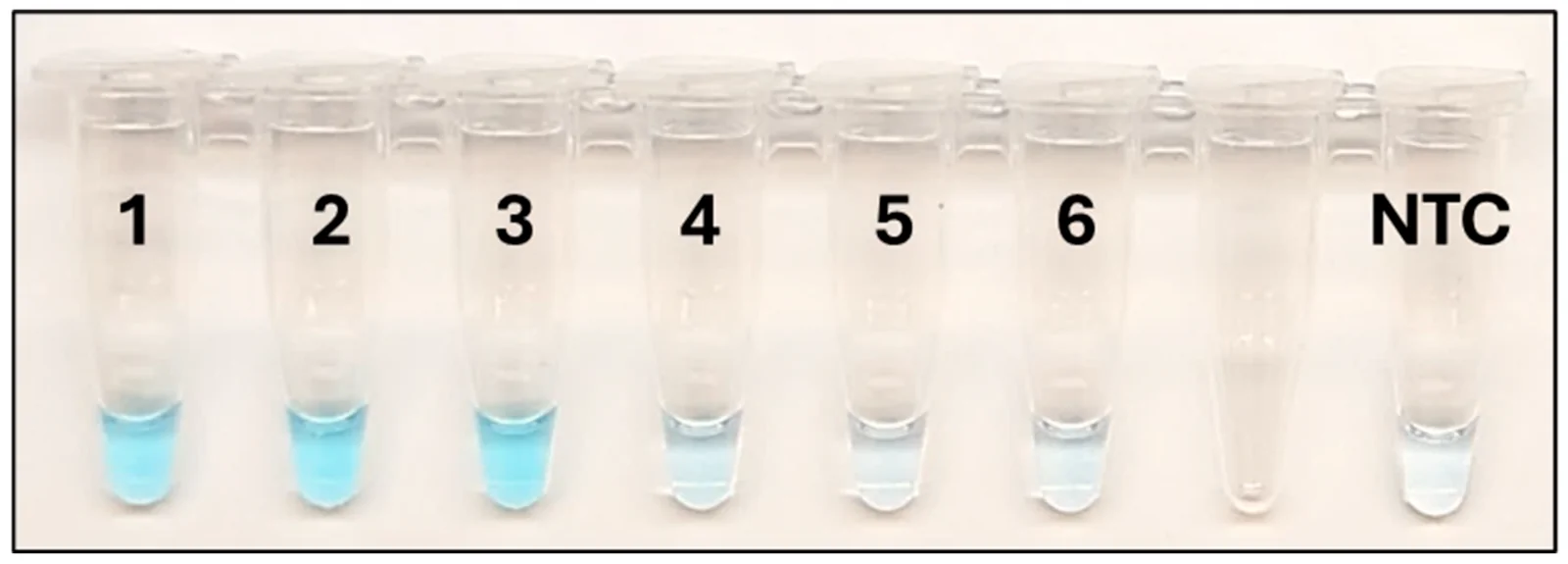
Small-scale diagnostic specificity and sensitivity testing results for the colorimetric LAMP assay (Assay 4). Diagnostic samples from the U.S. testing positive for *Ralstonia* spp. were previously tested. Positive results are viewed as a color change from clear (color before incubation) to blue. (1) 23030901 (I-13); (2) 17092702-04 (I-18); (3) 24082202-01 (I-30); (4) 24082802-01 (IIA-38); (5) 20041701-01 (IIB-1); (6) 24022201-01 (IIC-7); NTC = no-template control. Only *R. pseudosolanacearum* (Phylotypes I) samples produced positive results with the colorimetric LAMP assay.

A smaller-scale test panel was again used to corroborate diagnostic specificity and sensitivity in the portable instruments. Testing in portable instruments corroborated the 100% diagnostic specificity and sensitivity established using real-time PCR instruments. Only *R. pseudosolanacearum* (Assay 1) was detected and determined to be phylotype I (Assays 2 and 3) (Supplementary Table S5). The colorimetric assay (Assay 4) was also tested and revealed 100% specificity (Figure 2). As part of a PPQ Cooperative Agricultural Pest Survey (CAPS), symptomatic eggplant samples from Guam, as well as symptomatic eggplant and tomato from Hawaiʻi, were acquired for testing; *R. pseudosolanacearum* phylotype I is known to be present in Guam and Hawaiʻi. Plant samples were processed using the DNeasy^®^ Plant Mini Kit by streaming and then tested with LAMP and 16S PCR. All assays produced congruent positive results for eggplant, suggesting infection by a phylotype I strain, thus demonstrating the utility of these assays; results were confirmed using *egl* sequencing. The Hawaiʻi tomato sample stream tested negative by LAMP and 16S PCR; this sample was from a higher part of the stem on a sample already showing low titer (C_t_ ~35) with a kit extraction.

### 2.5. Assay Precision

A blind panel was prepared in an eight-well tube strip containing (1) 202402-02 (I-30), (2) UW685 (III-44) heat-lysed cells near the LoD, (3) Molk2 (IIB-3) DNA at 1 ng/µL, (4) LMG27703^T^ (IV-10) DNA at 1 ng/µL, (5) UW386 (III-23) DNA at 1 ng/µL, (6) CP synthetic control, (7) HP synthetic control, and (8) MG water; samples were tested in triplicate for each assay. At the time these tests were performed, PPCDL had three AmpliFire^®^ Isothermal Fluorometers and three Genie^®^ III’s; for a given assay, each replicate was run in a different instrument. All three operators produced congruent results (N = 9) across all assays and instruments, producing positive results for *R. pseudosolanacearum* only (Assays 1 and 4) and accurately identifying *R. pseudosolanacearum* phylotypes I and III (Assays 2 and 3) (Supplementary Table S6).

### 2.6. European Phytosanitary Research Coordination (Euphresco) Samples

**Figure 3:**
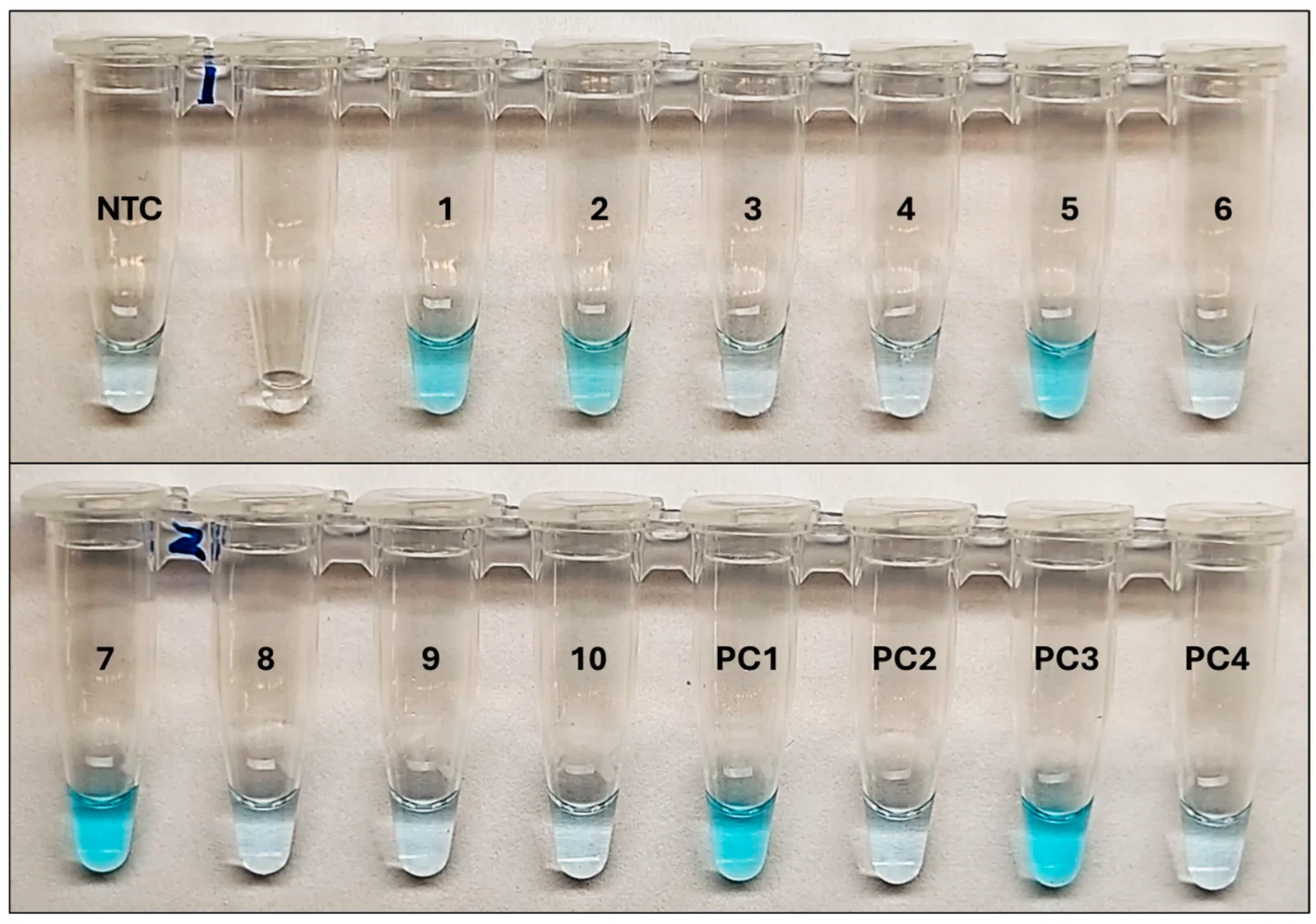
Euphresco sample results obtained with the colorimetric LAMP assay (Assay 4) using kit-extracted DNA. Ten blind samples and four positive controls (PC1–PC4) were provided as part of the Euphresco panel. Samples 1, 2, 5 and 7 produced positive results, as seen by a color change from clear (color before incubation) to blue. Additionally, PC1 and PC3 produced positive results. NTC = no template control. The assay accurately identified the blind samples, based on the revealed sample and control identities: (1) *Ralstonia pseudosolanacearum* (I, PD7123) in rose extract; (2) *R. pseudosolanacearum* (I, PD8383) in ginger rhizome extract; (3) *R. syzygii* subsp. *indonesiensis* (IV, R784); (4) surface water control; (5) *R. pseudosolanacearum* (I, PD8131) and *R. solanacearum* (II, PD7106) in surface water; (6) potato tuber extract control; (7) *R. pseudosolanacearum* (III, GBBC3272) in potato tuber extract; (8) *R. solanacearum* (II, PD7421) in rose stem extract; (9) *R. syzygii* (IV, MAFF301558) in potato tuber extract; (10) ginger rhizome extract control; PC1) *R. pseudosolanacearum* (I, PD7123); PC2) *R. solanacearum* (II, PD7106); PC3) *R. pseudosolanacearum* (III, GBBC3272); PC4) *R. syzygii* subsp. *indonesiensis* (IV, R784).

DNA extracted from 10 blind samples and four PCs were tested with all assays in their appropriate instruments. All assays produced congruent results for the kit-extracted samples, detecting and/or differentiating *R. pseudosolanacearum* phylotypes I and III in samples 1, 2, 5 and 7 (Table 1 and Figure 3); PC1 and PC3 were identified as *R. pseudosolanacearum* phylotypes I and III, respectively. Following submission of all participant data to the project organizers, sample and control identities were revealed. The LAMP assays accurately identified samples containing *R. pseudosolanacearum,* and Assays 2 and 3 were able to determine the appropriate phylotype (I or III).

### 2.7. Inoculated Plant Samples

At PPCDL, two inoculated geraniums and one inoculated potato developed symptoms; these were inoculated with UW685 (III-44). Symptomatic tissue produced positive results with all assays, and Assays 2 and 3 correctly identified the infecting phylotype as III; healthy (mock-inoculated) plants produced no symptoms and negative LAMP results. Several asymptomatic plants (two tomatoes and the other potato infected with UW685) tested positive by the LAMP assays. All positive and negative results were confirmed by 16S real-time PCR (Supplemental Table S7 and Supplementary Figure S2). The positive asymptomatic potato plant produced a weak positive with the colorimetric LAMP assay (Assay 4); this sample had a C_t_ value of ~36. Plants inoculated with 202402-02 (I-30) did not develop symptoms or produce positive results.

Rose varieties “Rio Samba” and Pink Double Knock Out^®^ failed to develop symptoms when infected with either strain. When tested with 16S PCR initially, three of six “Rio Samba” and five of eight Pink Double Knock Out^®^ roses inoculated with PD7123 produced positive results, while only one of six “Rio Samba” and one of eight Pink Double Knock Out^®^ roses inoculated with UW764 produced positive results (Supplementary Table S8); all mock-inoculated plants tested negative. All samples were low-titer, with C_t_ values of 30–36 and most being ≥34, and all tested negative by LAMP initially (Supplementary Table S8). During preliminary testing of one rose variety per strain, a single asymptomatic rose tested positive by the 16S assay; however, this sample produced negative results with the LAMP assays, suggesting inhibition. This sample was asymptomatic and low-titer (C_t_ value ~31). When the sample extract was cleaned using the DNeasy^®^ PowerClean^®^ Cleanup Kit (Qiagen), the LAMP assays produced positive results, confirming inhibition. Many of the inoculated-sample streams were dirty/dark-colored; mock-inoculated streams were generally clear. Experimental testing with this type of stream showed inhibition even with PerfeCTa^®^ qPCR ToughMix^®^; therefore, all samples were cleaned with the DNeasy^®^ PowerClean^®^ Cleanup Kit and retested (Supplementary Table S8). After cleaning, only two of six “Rio Samba” and four of eight Pink Double Knock Out^®^ roses inoculated with PD7123 produced positive results, while only two of six “Rio Samba” and two of eight Pink Double Knock Out^®^ roses inoculated with UW764 produced positive results (Supplementary Table S8).

For compatibility with LAMP, a different extraction method was tested to accommodate asymptomatic (putative low-titer) roses to remove inhibitors; refer to Section 3 and Section 4 for additional details. Rose streams testing positive by 16S PCR were “cleaned” using the alternate protocol and tested by 16S PCR and LAMP (Supplementary Tables S7 and S8). Unfortunately, inefficient recovery of the clean-up pushed most of the already low-titer samples below the limit of detection for the LAMP assays, as well as PCR; recovery issues were similar even with the commercial clean-up kit. The only sample able to show positive LAMP results after field clean-up was the single Pink Double Knock Out^®^ rose inoculated with UW764 that produced 16S PCR positive results initially; the C_t_ value of the cleaned sample was ~34. Rose variety Papa Meilland^®^, which was reported symptomatic in publication, also failed to develop symptoms when infected with PD7123. However, at least two inoculated plants produced 16S PCR-positive results for a few of the shoots sampled from these plants. All PCR-positive samples were cleaned prior to testing with LAMP. All cleaned samples produced positive results for all LAMP assays (Supplementary Table S7 and Figure S2).

At ARS, almost all inoculated tomatoes (27 of 32 total) produced symptoms within two weeks post-inoculation, for all inoculating strains (seven of eight for Rs121, six of eight for Pss262, six of eight for GMI1000 and eight of eight for RUN302); mock-inoculated plants remained asymptomatic. All tomatoes inoculated with phylotype I isolates produced strong positive results with the LAMP assays; mock-inoculated plants produced negative LAMP results (Supplementary Table S7 and Figure S2). Tomatoes inoculated with RUN302 (IIB-4) produced weak positive results by the LAMP assay. The time-to-positive was ~25 min, indicative of a low-titer infection. The RUN302 culture used for the inoculations was found to be contaminated by an unknown phylotype I strain and was deemed responsible for the observed results; refer to Section 3 for additional details.

### 2.8. Synthetic Control

The synthetic controls were tested individually for each assay at 100 ag/µL. Results showed both CP and HP were detectable by all assays. HP appeared to amplify faster than CP and showed a large difference in RFU in the Genie^®^ III (25 K for CP vs. 45 K for HP); the observed phenomenon was likely due to the increased probe concentration for the HP primer set and increased efficiency of target amplification due to the inclusion of two loop primers. The controls also produced positive results with the colorimetric assay, with CP producing weaker positives than HP, which corresponded to the increased time-to-positive observed with real-time assays. Based on control data, it is recommended that the HP control at 100 ag/µL be used for all assays. The CP control should be included as a separate second control with real-time Assays 2 and 3; controls need to be run separately, as including both in the same well will create a multiplex LAMP that will require optimization of components such as dNTPs and Mg^2+^ to allow simultaneous amplification of two distinct targets.

## 3. Discussion

*Ralstonia pseudosolanacearum* comprises two phylotypes (I and III) from different geographic origins. Phylotype I strains infect a broad range of economically important hosts and are distributed globally, including the U.S. Strains of *R. solanacearum* phylotypes IIA and IIB have also been found in the U.S.; however, phylotype IIB sequevars 1 and 2 remain exotic to the U.S. and warrant considerable concern. As a result, phylotype IIB sequevars 1 and 2 are designated as U.S. Select Agents and have heightened regulations. Any detection of *Ralstonia solanacearum* is treated as a potential Select Agent until shown otherwise. This revealed a need for a rapid test that could discriminate *R. pseudosolanacearum*, which occurs domestically, from Select Agent strains.

The availability of validated diagnostic tools is the cornerstone of successful surveillance, quarantine, and eradication measures. A literature search was performed and revealed several LAMP protocols for detection of *R. pseudosolanacearum* phylotype I, with varying degrees of specificity [28,35,36]; no assays were identified to detect phylotype III. The assays by Prameela et al. [35] and Huang et al. [28] targeted specific subpopulations of *R. pseudosolanacearum* (Race 4 and phylotype I mulberry strains, respectively), while the assay by Robène et al. [36] was designed to detect all strains of phylotype I. High specificity for phylotype I was demonstrated for the Robène et al. [36] assay; however, among the strains included in the study, two isolates from mulberry in China (UW373 and UW361) were not detected. It was unknown if other strains not included in the study would also produce false negatives; regardless, this assay was evaluated here.

An additional literature search was performed to identify target regions from other assays that can discriminate *R. pseudosolanacearum* and would make useful markers for LAMP primer design. Several publications were identified that reported discrimination of *R. pseudosolanacearum* (phylotypes I and/or III) [1,45,46,47]. Upon more detailed analyses, two assays targeted sequence differences within the 16S–23S ribosomal intergenic spacer region [1,46], while another assay targeted sequence differences in *egl* [47]. While these targets can work for PCR, they are present in all RSSC strains and make it difficult to identify sequence differences sufficient for proper LAMP primer design. The assay by Paudel et al. [45] was the only one presenting a target unique to *R. pseudosolanacearum*, an intergenic region between a Flp family type IVb pilin and a TPM (Thioredoxin-like fold, Phosphatase, and Metalloenzyme) domain-containing protein. High specificity for *R. pseudosolanacearum* (phylotypes I and III) was demonstrated; however, among the strains included in the study, two phylotype III isolates (JT525 and NCPPB332) were not detected. It was unknown if other strains not included in the study would also produce false negatives; regardless, LAMP primers were designed to this target and evaluated in this study.

Assays by Paudel et al. [45] and Robène et al. [36] both produced false negative results for known *R. pseudosolanacearum* isolates, and no specific targets were found for phylotype III. Thus, *Ralstonia* genomes were compared, and novel targets were identified for phylotypes I and/or III. Several target genes were identified that would detect both phylotypes (*R. pseudosolanacearum*) theoretically, but these genes were unsuitable for LAMP primer design due to single nucleotide polymorphisms (SNPs) present between the two phylotypes. PCR primers and probes were designed for two of these target genes (putative outer membrane protein (porin) and putative NADPH nitroreductase), as conserved regions were identified that could be exploited as primer binding sites. Target genes and primer binding sites were checked for in silico specificity, and results showed that these potential assays should detect all *R. pseudosolanacearum* isolates that have sequence data available in GenBank. However, these were not investigated further as this study was to develop a portable assay, but PCR primers and probe options are available for future testing. Additionally, many other targets were identified but eliminated from further investigation due to the inability to design LAMP primers.

Several phylotype-specific target genes were identified following additional analysis; four were identified as candidates for phylotype III detection, and one was identified as a candidate for phylotype I detection. LAMP primers were designed for these target genes and tested. Preliminary results showed that the phylotype I-specific target (CP) and one phylotype III-specific target (HP) were sufficient candidates for further evaluation. The Robène et al. [36] assay was tested alongside CP and HP primer sets, as well as primer sets designed to the Paudel et al. [45] target. Preliminary results showed that LAMP primers for the Paudel et al. [45] target and the Robène et al. [36] assay produced false-negative results in this study for the same reported strains. The LAMP primers for the Paudel et al. [45] target also had an increased time-to-positive, which resulted in decreased sensitivity; therefore, this assay was not evaluated further. Phylotype I-specific (CP and Robène et al. [36]) and phylotype III-specific (HP) primer sets were mixed together in a multiplex assay format. For real-time application, probes were designed for each target primer set. Phylotype I probes were labeled with FAM and the phylotype III probe was labeled with either FAM (single FAM output to detect *R. pseudosolanacearum* I and III) or another instrument-specific fluorophore (e.g., HEX and ATTO590 for the AmpliFire^®^ Isothermal Fluorometer and Genie^®^ III, respectively) to allow differentiation of phylotypes I and III. Initial tests of the duplexed assays showed that CP and HP were more compatible and produced better results. Thus, the Robène et al. [36] assay was not evaluated further.

The assays developed in this study demonstrated high sensitivity when using heat-lysed cell suspensions; much better than with kit-extracted DNA. Sensitivity was similar to results produced by the 16S PCR assay for kit-extracted DNA. Multiple copies of the 16S rRNA gene exist between the chromosome and megaplasmid, with most strains having four copies, while the specific LAMP targets exist in single copy. The polymerase in ISO-004nd has strong reverse transcriptase activity, so it was hypothesized that the inclusion of RNA in the heat-lysed cell extracts was responsible for the increased sensitivity. However, multiple experiments testing addition of RNase A to crude extract to remove RNA failed to show any significant differences. It is possible that this, in combination with the fact that LAMP reagents may perform optimally with cruder input, is responsible for the observed sensitivity; however, the true reason for this observed phenomenon remains unknown/unproven at this time. The assays were also tested briefly using a lyophilized version of the LAMP master mix (ISO-DR004nd, OptiGene Ltd.). Preliminary tests with the lyophilized reagent produced poor results, suggesting a potential decrease in sensitivity. Thus, the use of the lyophilized reagent was not explored further in this study but could be revisited in the future.

The CP and HP targets demonstrated 100% specificity for *R. pseudosolanacearum* and were able to differentiate phylotype I and III strains, respectively, with one exception. Phylotype III sequevar 23 strains contain both target genes. When CP and HP were tested individually, a strong positive signal was observed for each target; however, the validated duplexed assays performed differently. At lower concentrations (near the LoD), signals for both targets were seen occasionally. At higher concentrations, the assays appeared to favor the phylotype III-specific marker; sequevar 23 strains are designated phylotype III, so this is the correct determination. For example, phylotype III strain CFBP3059 was tested at ≤0.05 ng/µL (≤0.25 ng/reaction) and showed positive results for both targets, while phylotype III strain UW386 was tested at 1.0 ng/µL (5 ng/reaction) and only produced positive results for phylotype III. When CFBP3059 concentrations were increased to ~1.0 ng/µL, only phylotype III signal was observed. Phylotype III sequevar 23 strains appear rare [48] and, in practice, testing symptomatic tissue should provide sufficient DNA such that only signal from the phylotype III marker will appear for sequevar 23 strains.

Nine phylotype II strains produced positive signal with the phylotype I LAMP marker. There was no pattern among the strains regarding sequevar, host, or geographic location. The late signals produced by these strains were similar to those observed with low-titer positive samples, suggesting the presence of low-level phylotype I contamination in either the strains themselves or the extracted DNA. Of these nine strains, three have published genomes available (RUN302, UW349 and UW425), and a fourth strain (UW501) appeared highly similar to UW500, which has a published genome, is from the same host and geographic location, is named sequentially, and tested negative by the LAMP assays. Sequence analysis revealed that no LAMP amplicons should be produced for these strains, as evidenced by the absence of proximal primer and probe binding sites within the genomes. DNA extracted from cultures of these phylotype II strains was shown to contain phylotype I, based on cPCR using phylotype-specific primers [1,45]. Strain UW425 produced the fastest LAMP signal and had the brightest band, indicating the highest relative concentration of phylotype I. Additionally, strains RUN302, RUN141 (CFBP7027) and RUN160 (JT516) were shown to test negative by a published phylotype I-specific LAMP assay [36]. The Robène et al. [36] LAMP assay was used to test DNA provided for these three strains and produced positive amplification, contradicting published results for these strains and thereby suggesting the presence of phylotype I. These results were able to show true contamination of the phylotype II strains (UW425 and RUN302) with an unknown phylotype I strain. Two cPCR assays and a published LAMP assay corroborated the LAMP results produced in this study, further demonstrating the specificity and sensitivity of the assays presented in this study. The cPCR assays were also used to test tomato samples inoculated with RUN302 and revealed the same low-level contamination from a phylotype I strain. The source of this contamination is unknown. Contamination may have arisen from a mixed infection in the original samples used for strain isolation or from incomplete purification of the strains during the isolation process. Given the widespread global distribution of phylotype I strains, colocalization with phylotype II strains is possible, even if they may not be the predominant phylotype in those areas.

During specificity testing, one sample produced unusual results: *Dickeya solani* Ds0432.1. Amplification signal began immediately and increased in a linear-like manner; this was observed on multiple machines and produced false-positive result calling. The original sample was analyzed and found to contain ~182 ng/µL DNA, which means the DNA input per reaction was near 1 µg; this was very high but not suspected to be the cause of the issue. It was discovered that this sample was a heat-lysed-cell preparation from cells stored with an unknown concentration of DMSO; DMSO can be used as a cryoprotectant. As DMSO can denature DNA, it was hypothesized that DMSO was denaturing the probe-quencher complex, thus creating a positive signal. Dilutions of the sample showed that the positive signal decreased with each dilution, and at a 1:20 dilution (~10 ng/µL), the positive signal was removed such that the AmpliFire^®^ Isothermal Fluorometer was able to recognize it as a negative (Supplementary Figure S3). Additionally, at this concentration, the DNA input for the reaction was ~50 ng. This amount of input is sufficient to conclude that the signal was not a cross-reaction due to high concentration, and that the loss of signal was not due to simply diluting out the DNA. Moreover, the original sample tube and 1:20 and 1:200 dilutions were tested with the colorimetric LAMP assay and produced negative results (Supplementary Figure S4). This suggests that the signal observed with the real-time assay was not due to amplification of target DNA, but simply a result of signal production due to probe displacement from the quencher. A DNA extraction was performed on the same isolate from fresh culture, and the DNA tested negative by all assays, confirming assay specificity.

The assays developed in this study also demonstrated 100% diagnostic sensitivity and specificity when tested using diagnostic samples confirmed positive for *Ralstonia* by PPCDL. The majority of these samples originated in the U.S. mainland and Guam and represented RSSC strains that could be encountered during surveys, thus providing an accurate evaluation of diagnostic specificity and sensitivity. Samples positive for *R. solanacearum* Phylotype IIB sequevar 1 resulted from an incursion; *R. solanacearum* phylotype IIB sequevar 1 was eradicated in the U.S. following the incursion [49]. One sample from pepper in Guam (17092702-07) produced a 16S C_t_ value of ~36. This low-titer sample was detectable by LAMP Assays 1 and 2 in real-time PCR instruments, as well as by Assay 1 in both the AmpliFire^®^ Isothermal Fluorometer and Genie^®^ III. This sample was not tested with the other assays as the DNA was exhausted during testing; this sample was from 2017 and used in other *Ralstonia*-related projects. This data further supports the high sensitivity and practical applicability of these assays for testing in the field.

The assays were also used to test a panel of blind samples provided to PPCDL for a Euphresco project. When using kit-extracted DNA, the assays were able to correctly identify all *R. pseudosolanacearum* samples in the panel, and Assays 2 and 3 were able to discriminate phylotypes I and III. The sample panel was also tested in crude form by simply incubating samples at 95 °C for 5 min. Samples produced the same results for heat-lysed cells as with kit-extracted samples, except for Sample 1. Samples 1 and 8 were highly colored (rusty red color). These samples were rose stem extracts and likely contained too many inhibitors in the crude extract; the samples appeared to contain macerated tissue. Inhibition in Sample 1 was also seen with kit-extracted DNA when comparing results from real-time [50] and conventional [1] PCR assays used for the Euphresco ILC.

The protocol described here for processing plant samples exploits bacterial streaming from infected material to minimize host inhibitory compounds; bacterial streaming was considered a preliminary test for *Ralstonia* due to the high amount of white bacterial ooze present in infected tissue [51]. This study showed that *R. pseudosolanacearum* phylotypes I and III can be detected from a variety of hosts when using the streaming method to produce a crude extraction. However, low-titer asymptomatic roses failed to produce positive results; this was attributed to inhibition. Rose varieties “Rio Samba” and Pink Double Knock Out^®^ were very difficult to infect, with only a few plants producing 16S PCR positive results that reflected low titers or cross-reactions from the assay. Papa Meilland^®^ roses also failed to produce symptoms, but two plants were able to produce 16S PCR-positive results for some of the harvested shoots with C_t_ values in the mid-to-late 20s. These assays were intended to be used for testing symptomatic plants; thus, this does not detract from their utility. Additionally, a DNA clean-up kit was able to remove inhibitors and allow positive results from the LAMP assays; however, requiring a clean-up kit precludes practical field application.

A way to combat inhibition in roses was investigated by adapting the DNeasy^®^ PowerClean^®^ Pro Cleanup Kit for the field. Streams (400 µL) of healthy rose stems were spiked with ~20,000 GECs 202402-02 (phylotype I) (40 µL of ~500 GEC/µL, ~C_t_ 25). The spiked stream was processed using a modified cleanup protocol (100 µL input) that eluted in 100 µL TE buffer, resulting in samples containing ~45 GEC/µL, which corresponds to ~225 GEC/reaction. The field clean-up protocol enabled positive LAMP results; LAMP assays were negative before clean-up. When compared to the manufacturer’s protocol, 16S PCR C_t_ values were ~32 and ~30 for the field and lab protocols, respectively, revealing a slight loss in recovery, which could lead to false negative results for very low-titer samples. However, the protocol did demonstrate the ability to clean rose streams and allow compatibility with the LAMP assays. The loss in sensitivity does not detract from its utility in allowing testing of difficult samples such as roses that contain a lot of inhibitors. When tested using inoculated rose samples, the field clean-up protocol allowed positive LAMP results from rose streams testing negative prior to clean-up; results corroborated PCR results. Experiments using rose streams spiked with increased amounts of *R. pseudosolanacearum* cells were able to show that inhibition can be overcome with higher titer samples, resulting in LAMP-positive results. However, it is unknown how this relates to natural infection in roses, particularly when symptoms appear. It is possible that symptomatic roses will have sufficient titers to overcome inhibition, but this information is unknown at this time; thus, the clean-up method is recommended for roses at this time. Additionally, the clean-up method can be applied to other samples if inhibition is concerning, such as with dirty and/or highly colored samples.

The cost per reaction for Assays 1–4 is $2.11 USD, $2.20 USD, $2.41 USD, and $1.85 USD, respectively, based on the reagents and oligonucleotides used per reaction, plus a 20% increase to account for disposable supplies like tips, tubes, and gloves; this does not reflect operational costs. The cost of using the synthetic gBlocks^TM^ positive control was also calculated based on rehydration and dilution to 100 ag/µL in 0.5 mg/mL poly(A) in TE buffer. The cost for every 1000 reactions was calculated to be $8.27 USD for each control; the largest contributing factor to cost was the poly(A) and then the TE buffer. This equates to less than 1¢ per reaction per control; cost calculations do not reflect operational costs. The cost per sample when using the field extraction was calculated to be ~$8.99 USD, based on the use of a specific brand and number of filters and syringes; use of a different brand and/or number of syringes and filters could increase costs. The calculated cost does not reflect the operational costs to perform the extractions, nor does it include the one-time cost of the instrument to help dry the membranes; the WOLFBOX MF50 Electric Air Duster was $39.99 each.

## 4. Materials and Methods

### 4.1. Strains, Plant Hosts and DNA Extraction

The 230 bacterial strains used in this study are shown in Supplementary Table S2. DNA for most of these organisms was obtained from various sources, being provided generously to the USDA APHIS PPQ Science and Technology (S&T) Plant Pathogen Confirmatory Diagnostics Laboratory (PPCDL) by collaborators or purchased from culture collections. RSSC isolates (N = 177) comprised all three plant-pathogenic *Ralstonia* species and all four phylotypes, including the type strains: 81 phylotype I (*R. pseudosolanacearum*), 70 phylotype II (*R. solanacearum*), 15 phylotype III (*R. pseudosolanacearum*), and 11 phylotype IV (*R. syzygii*). Additionally, DNA from the type strains of three *Ralstonia* species isolated from humans, and another *Ralstonia* species isolated from soil, was included in this study. Forty-nine additional non-*Ralstonia* bacterial pathogens from relevant hosts and soil were included in this study. For any bacterial isolates obtained or purchased as cultures, DNA was extracted using the DNeasy^®^ Plant Mini Kit and/or DNeasy^®^ Blood & Tissue Kit (Qiagen, Germantown, MD, USA), according to the manufacturer’s instructions.

Healthy planting material was purchased from commercial sources and then propagated in greenhouses at PPCDL. DNA was extracted from healthy pepper (*Capsicum annuun*), oleander (*Nerium oleander*), geranium (*Pelargonium zonale*), tomato (*Solanum lycopersicum*), eggplant (*Solanum melongena*), potato (*Solanum tuberosum*), rose (*Rosa* sp.), blueberry (*Vaccinium* sp.), and ginger (*Zingiber officinale*), using the DNeasy^®^ Plant Mini Kit with minor modifications. Briefly, 100 mg of tissue was processed with 600 µL AP1 buffer and 6 µL RNase A (100 mg/mL) in a Lysing Matrix A tube with an additional ceramic bead (MP Biomedicals, Solon, OH, USA) using a FastPrep-24™ 5G bead-beating grinder and lysis system (MP Biomedicals) for two runs at a speed setting of 6.0 for 40 s each. Tubes were incubated in a thermomixer at 65 °C for 10 min with 300 rpm agitation. Following incubation, 195 µL Buffer P3 was added to each tube, followed by vortexing and incubation on ice for at least 5 min. The remaining steps of the extraction were carried out according to the manufacturer’s instructions.

Fresh strawberries (*Fragaria* × *ananassa*) (Driscoll’s, Watsonville, CA, USA) and freeze-dried strawberry fruit tissue (Brothers All Natural, Rochester, NY, USA) were purchased from a local grocery store. DNA was extracted from calyx or fruit tissue using the DNeasy^®^ Plant Mini Kit with minor modifications. Briefly, 100 mg of tissue was processed with 800 µL AP1 buffer and 8 µL RNase A (100 mg/mL) in a Lysing Matrix A tube with an additional ceramic bead (MP Biomedicals) using a FastPrep-24 (MP Biomedicals) for two runs at a speed setting of 6.0 for 40 s each. Tubes were incubated in a thermomixer at 65 °C for 10 min with 500 rpm agitation. Following incubation, 260 µL Buffer P3 was added to each tube, followed by vortexing and incubation on ice for at least 5 min. The remaining steps of the extraction were carried out according to the manufacturer’s instructions.

### 4.2. Target Identification and Primer Design

Whole-genome sequences for 12 RSSC strains (phylotypes I-IV), as well as 288 closely related species and genera, were acquired from the National Center for Biotechnology Information (NCBI) GenBank (Supplementary Table S9). Genomes were annotated and compared to identify orthologous genes using the MicroScope Microbial Genome Annotation and Analysis Platform v3.17.3 [39,40,41,42,43,44] under stringent parameters (80% amino-acid identity, 80% amino-acid alignment coverage). Potential diagnostic target sequences were evaluated for *R. pseudosolanacearum* specificity using BLASTn v.2.12.0 [52] comparisons against a local database of 375 *Ralstonia* genomes curated from NCBI.

LAMP primer sets were designed to the phylotype I-specific (conserved protein (CP) of unknown function) and phylotype III-specific (hypothetical protein (HP)) markers using PrimerExplorer V5 (Eiken Chemical Co. Ltd., Tokyo, Japan) (https://primerexplorer.eiken.co.jp/lampv5e/index.html, accessed on 7 October 2026) and evaluated using NCBI Primer-BLAST (https://blast.ncbi.nlm.nih.gov/Blast.cgi, accessed on 7 October 2026) [52] to assess matches with non-targets, with default parameters. Primer binding sites were mapped to target sequences in Geneious Prime^®^ 2023.0.4 [53], and the target amplicons were extracted and rescreened using NCBI BLASTn (megablast and blastn with default parameters) to assess matches to non-targets; whole-genome shotgun contigs (wgs), core nucleotide (core_nt), Refseq Genome (refseq_genomes), and Refseq Reference Genome (refseq_reference_genomes) databases were queried. Primer and probe sequences used in this study are shown in Table 2.

### 4.3. LAMP Reaction Conditions

Four different assays were developed and validated in this study: (1) real-time LAMP for detection of all *R. pseudosolanacearum* (I and III) using the AmpliFire^®^ Isothermal Fluorometer (Agdia Inc., Elkhart, IN, USA) or Genie^®^ III (OptiGene Ltd., West Sussex, UK); (2) and (3) real-time LAMP for detection and differentiation of *R. pseudosolanacearum* phylotypes I and III in the AmpliFire^®^ Isothermal Fluorometer or Genie^®^ III, respectively; (4) colorimetric LAMP for detection of all *R. pseudosolanacearum* strains. Real-time LAMP assays consisted of 15 µL GspSSD2.0 Isothermal Mastermix without dye (ISO-004nd) (OptiGene Ltd.), 5 µL primer mix (pMix), and 5 µL template in a final volume of 25 µL. Colorimetric LAMP reactions consisted of 15 µL GspSSD2.0 Isothermal Mastermix—VISUAL color change (ISO-004-VIS) (OptiGene Ltd.), 5 µL pMix, and 5 µL template in a final volume of 25 µL. Each pMix was a duplex containing CP and HP primer sets. For real-time assays, FAM-, HEX-, and ATTO590-labeled HP probes were used for Assays 1, 2, and 3, respectively; see Supplementary Tables S10–S13 for full pMix compositions of Assays 1–4, respectively.

All assays were run for 30 min. For the AmpliFire^®^ Isothermal Fluorometer, reaction conditions and analysis parameters were programmed into QR codes using AmpliCode™ (Agdia Inc.) (https://amplicode.agdia.com/, accessed on 7 October 2026); the QR codes are scanned by the instrument to set up a run (Supplementary Figure S5). For the Genie^®^ III, melt curve analysis was disabled, and analysis parameters were updated: Type: Threshold, Plot: Normalized, Range X 180 to 1800 s, and Range Y 3500 to 3500 F for Channel 1 (Assays 1 and 3) and Channel 2 (Assay 3 only). Additionally, result output settings were changed such that the instrument would display “Positive” and “Negative” for corresponding results based on curve analysis, thus eliminating user interpretation. Colorimetric LAMP assays were run in a ProFlex PCR System (Applied Biosystems, Waltham, MA, USA) for 30 min at 65 °C, followed by 2 min at 85 °C to inactivate the polymerase and stop the reaction for normalization of results; this step is not necessary in practice, and the assay can be run in a simple heat block capable of holding 65 °C.

The AmpliFire^®^ Isothermal Fluorometer and Genie^®^ III accommodate a single 8-well strip tube. For large-scale tests, Assay 1 was run in the QuantStudio™ 5 Real-Time PCR System (Applied Biosystems) and Assay 2 in the CFX Opus Real-Time PCR System (Bio-Rad, Hercules, CA, USA). For the QuantStudio™ 5 Real-Time PCR System, reactions were run according to the following conditions: a hold stage at 65 °C for 1 s and a PCR stage of 60 cycles at 65 °C for 30 s. A ramp rate of 3.66 °C/s was used for each stage, and the Passive Reference was set to None. For the CFX Opus Real-Time PCR System, reactions were run for 60 cycles at 65.0 °C for 20 s with Data Collection ON; the instrument was set to read all channels.

### 4.4. PCR Reaction Conditions

A real-time PCR assay targeting the 16S rRNA gene and designed to detect all RSSC strains [55] was used in this study to verify and quantify DNA extractions and dilutions. Primers and probe sequences are shown in Table 2. Reactions contained 1X PerfeCTa^®^ qPCR ToughMix^®^ Low ROX™ (Quantabio, Beverly, MA, USA), 240 nM primers, and 120 nM probe in a final volume of 25 µL. Template was adjusted to 5 µL to reflect the input for LAMP reactions for direct comparison. Reactions were run in the QuantStudio™ 5 Real-Time PCR System according to the following cycling conditions: initial denaturation at 95 °C for 2 min, followed by 40 cycles of 95 °C for 1 s and 60 °C for 40 s. When DNA was cleaned with the DNeasy^®^ PowerClean^®^ Pro Cleanup Kit (Qiagen), the master mix was switched to Luna^®^ Universal Probe qPCR Master Mix (New England BioLabs, Ipswich, MA, USA), as PerfeCTa^®^ qPCR ToughMix^®^ Low ROX™ was designed to combat inhibitory compounds, which are now absent from the DNA preparation.

### 4.5. Synthetic Positive Control

Two synthetic gBlocks™ (IDT) positive controls, CP and HP, were designed for use with the described LAMP assays (Supplementary Table S14). Each gBlocks™ was 250 bp in size and contained sequences for the two targets used to detect *R. pseudosolanacearum* and differentiate Phylotypes I and III. The CP control consisted of the 207 bp CP LAMP amplicon, plus an additional 20 bp upstream and 23 bp downstream. The HP control consisted of the 193 bp HP LAMP amplicon, plus an additional 24 bp upstream and 33 bp downstream. The controls were tested at ~100 ag/µL (~370 copies/µL) in 1X TE buffer (Promega, Madison, WI, USA) with 0.5 mg/mL polyadenylic acid (poly(A)) (Roche Diagnostics, Mannheim, Germany).

### 4.6. Plant Inoculations

Healthy geranium (“Americana Red Dark”) (Plantpeddler, Cresco, IA, USA), tomato (“Floradade”) (Eden Bros., Arden, NC, USA), pepper (“Green Machine”) (Seminis, St. Louis, MO, USA), potato (“Burbank Russet”) (Sprout Mountain Farms, Kingsport, TN, USA) and ginger (local grocer) were propagated in a PPCDL greenhouse. Potato, geranium, and ginger were grown in 6” pots, and tomato and pepper in 4” pots; plants were inoculated at age 6 mo, 18 mo, 18 mo, 5 wk, and 5 wk, respectively. Phylotype I (202402-02) and phylotype III (UW685) cultures were grown on casamino acid-peptone-glucose (CPG) agar [56] for ~48 h. Suspensions in water were made at ~10^8^ CFU/mL and quantified by OD_A600_ (=0.1) using the NanoDrop OneC (Thermo Fisher Scientific, Waltham, MA, USA). Plant roots were wounded by inserting a sterile scalpel into the soil approximately 1” from the crown. Cell suspensions (50 mL for 6” pots and 30 mL for 4” pots) were poured into the pots at the wounded area (N = 2 each, per strain); plants were slightly water-starved before inoculation to encourage uptake of bacterial suspensions. A set of mock-inoculated plants were prepared using sterile water in place of bacterial suspensions (N = 2 each).

Healthy roses (“Rio Samba” and Pink Double Knock Out^®^) were purchased from different nurseries (High Country Roses, Broomfield, CO, USA, and Starks Bro’s, Louisiana, MO, USA, respectively). Two rose isolates (PD7123 and UW764) were grown on 2,3,5-triphenyl-2H-tetrazolium chloride (TZC) agar [56,57] for ~48 h at 28 °C. Suspensions in sterile water were made at ~10^8^ CFU/mL and quantified by OD_A600_ (=0.1) using the NanoDrop OneC. Plant roots were wounded by inserting a sterile scalpel into the soil approximately 1” from the crown. Cell suspensions (50 mL) were poured into the pots at the wounded area (N = 6–8 each, per rose variety per strain). Plants were slightly water-starved before inoculation to encourage uptake of bacterial suspensions. Mock-inoculated plants were prepared using sterile water in place of bacterial suspensions (N = 3–4 per rose variety). All plants were grown in a temperature-controlled greenhouse (ambient ~25 °C) with a 12 h photoperiod.

Healthy bare-root Papa Meilland^®^ roses (Edmund’s Roses, Randolph, WI, USA) were also purchased and allowed to grow for 30 days. Rose isolate PD7123 was grown in BG broth (10 g/L bacto peptone, 1 g/L casamino acids, 1 g/L yeast extract, and 5 g/L glucose) [58] for 24 h at 28 °C, with shaking at 180 rpm. Suspensions were normalized to ~10^8^ CFU/mL and quantified by OD_A600_ (=0.1) using the NanoDrop OneC. Plant roots were wounded by inserting a sterile scalpel into the soil approximately 1” from the crown. Cell suspensions (50 mL) were poured into the pots at the wounded area (N = 8). To further facilitate infection and potential symptom development, a single petiole per stem was wounded, and 20 µL cell suspension was applied to the wound. Mock-inoculated plants were prepared using sterile water in place of bacterial suspensions (N = 2). All plants were grown in a temperature-controlled greenhouse (ambient ~25 °C) with a 12 h photoperiod.

At ARS, tomato seeds (‘Bonny Best’) (Park Seed, Hodges, SC, USA) were sown in Sun Gro^®^ Sunshine^®^ Mix #5 (Sun Gro^®^ Horticulture, Agawam, MA, USA) and grown on a mist bench in a greenhouse section at 22 °C with 14 h of light daily until germination. Plants were moved to a different greenhouse section and kept between 24 °C (night) and 30 °C (day) with 14 h of light daily. Once seedlings developed 2–3 true leaves (approximately two to four weeks post-germination), they were transplanted into 5-in pots containing Sun Gro^®^ Sunshine^®^ Mix #1. Seedlings were maintained for an additional 2–4 wks until inoculation. *Ralstonia pseudosolanacearum* phylotype I strains GMI1000, Pss262, and Rs121, and *R. solanacearum* strain RUN302 (phylotype II strain used as a control), were streaked from frozen stock onto TZC agar and incubated for 24 h at 28 °C. A single colony was selected and grown overnight in modified casamino acid peptone glucose medium [57,59,60] at 28 °C with shaking at 150 rpm. Suspensions in sterile water were made at ~10^8^ CFU/mL and quantified by OD_A600_ (=0.1) using the NanoDrop OneC. Plants were inoculated using the soil drenching method by applying 50 mL of cell suspension to each pot (N = 8 per strain). A set of mock-inoculated plants was prepared using sterile water in place of bacterial suspensions (N = 8 each).

### 4.7. Plant Sample Preparation

Inoculated plants at PPCDL were sampled following symptom development or after 8 wks post-inoculation (dpi) regardless of symptom development. At ARS, inoculated tomatoes were sampled 7 dpi; most plants were symptomatic. Symptomatic tissue was cross-sectioned (e.g., at the crown) using a clean disposable razor blade, and another cross-section was made approximately 2″ above the original cut to make a 2″ tissue segment. This was done to make manageable tissue samples that could be removed from tubes for further processing; thick tissue samples were trimmed to allow them to fit into a microcentrifuge tube. The lower portion of the symptomatic plant tissue was submerged in 400 µL TE buffer pre-aliquoted into a 1.5 mL microcentrifuge tube. Samples were incubated for 5 min at room temperature to allow bacterial streaming, then plant tissue was removed. Samples were then incubated at 95 °C for 5 min, and tubes were placed on ice for at least 2 min.

All rose varieties were asymptomatic and processed as described above. Rose samples demonstrated inhibition with the streaming method; therefore, an alternative field extraction method incorporating the DNeasy^®^ PowerClean^®^ Pro Cleanup Kit was used to remove inhibitors from the streams. Samples were streamed and heat-lysed as described above, then 100 µL stream was mixed with 50 µL Solution CU and 50 µL Solution IR in a disposable container (e.g., weighing boat); a pipette was used to add sample and buffers, and a 1 mL Luer Lock syringe was used for mixing. Next, 400 µL Solution SB was added; a pipette was used to add sample and buffers as well as for mixing solutions in small volumes. The mixture was aspirated with the same 1 mL Luer Lock syringe and then plunged through a 13 mm glass fiber VWR^®^ Syringe Filter (1.0 µm) to capture DNA. Approximately 1 mL of air was plunged at least twice to expel as much liquid as possible; this step was done following each liquid plunge through the filter. Using a new Luer Lock syringe, the filter was washed twice by plunging 500 µL Solution CB through the filter. Filters were dried using a handheld portable blower capable of ≥60,000 rpm (e.g., MegaFlow 50, Wolfbox, Denver, CO, USA). At 110,000 rpm and an ambient temperature of ~20 °C, 5 min was sufficient to dry the membrane; increased ambient temperature and decreased relative humidity can expedite this process. To elute DNA, 100 µL TE buffer was aspirated into a new 1 mL Luer Lock syringe and then plunged through the cleaned and dried capture filter. A graphical schematic of the DNA clean-up workflow is shown in Supplementary Figure S6.

### 4.8. Assay Validation Testing

Validation testing was performed to evaluate the analytical specificity and sensitivity, diagnostic sensitivity and specificity, repeatability, and precision of the optimized LAMP assays. DNA from known strains of *R. pseudosolanacearum* (phylotypes I and III) was used to determine analytical specificity (inclusivity) (Supplementary Table S2). DNA from known strains of *R. solanacearum* (phylotype II), *R. syzygii* (phylotype IV), non-plant-associated *Ralstonia* spp., and additional non-*Ralstonia* bacterial pathogens from relevant hosts and soil were also used to determine analytical specificity (exclusivity) (Supplementary Table S2). Additionally, DNA from healthy pepper, oleander, geranium, tomato, eggplant, potato, rose, blueberry, and ginger were used to determine analytical specificity (selectivity). Thirty-four diagnostic samples that tested positive for *R. pseudosolanacearum* (N = 22) or *R. solanacearum* (N = 12) previously by PPCDL diagnosticians were used to determine diagnostic specificity and sensitivity (Supplementary Table S4). Inoculated and mock-inoculated plants were tested to further assess diagnostic specificity and sensitivity.

DNA extracted from phylotype I (202402-02) and phylotype III (UW386, UW472) cultures were diluted in molecular-grade (MG) water to starting concentrations of ~1 ng/µL. Ten-fold serial dilutions were then made using MG water to determine assay analytical sensitivity; half-log dilutions were also made between the last three 10-fold dilutions. Approximate genome equivalent copies (GEC) per reaction were calculated based on the approximate average genome size of *Ralstonia* (5.5 Mb) using the following formula, where **X** = the amount of DNA (ng) and **N** = length of dsDNA (bp): $number\;of\;copies\;\left(molecules\right)=\frac{X\;ng*6.0221\times 10^{23}\frac{molecules}{mole}}{\left(N*660+\frac{g}{mole*bp}\right)*1\times 10^{9}\frac{ng}{g}}$.

Additionally, bacterial suspensions of 202402-02 (phylotype I) were made in TE buffer to an OD_A600_ = 0.1 (~10^8^ CFU/mL), measured using the NanoDrop OneC. Cells were diluted in TE buffer to ~500 CFU/µL, followed by 1:2 dilutions in TE down to ~0.5 CFU/µL. Cell dilutions were incubated at 95 °C for 5 min and then placed on ice for at least 5 min before testing. For UW685 (phylotype III), dilutions were also made in TE buffer, but only dilutions near the limit of detection (LoD), as established for 202402-02 (phylotype I), were tested. The determined LoD was further tested in different host background exudates. Healthy geranium, oleander, rose, and tomato exudates were used as diluents to make cell dilutions near the LoD.

Intermediate precision (N = 9) was evaluated using data from three operators from different laboratory teams at USDA APHIS PPQ S&T PPCDL. Pre-tested reagents were provided to participants, along with a copy of the testing protocol. A blind test panel was also provided and consisted of eight samples, including controls, that were tested in triplicate using all described assays.

All assay results were binary, with positive results based on the presence of an appropriate amplification curve for real-time assays or an appropriate color change for the colorimetric assay; negative results were the absence of curves/color change. Analysis parameters were programmed into the portable instruments, and result outputs were +/− or Positive/Negative for the AmpliFire^®^ Isothermal Fluorometer and Genie^®^ III, respectively; no interpretation required.

### 4.9. Euphresco Samples

PPCDL participated in a Euphresco ILC (Interlaboratory Comparison) on the diagnostics of *R. pseudosolanacearum* and *R. syzygii* (project 2023-F-431 ExoRal). A panel of 10 unknown samples and four positive control (PC) samples were sent to PPCDL from the Netherlands Institute for Vectors, Invasive plants and Plant health (NIVIP). DNA was extracted from supplied crude samples and controls using the DNeasy^®^ Plant Mini Kit; the modified protocol stated above for healthy plant tissue was used, and the 100 mg tissue was substituted with 100 µL crude extract. Additionally, samples and controls were incubated at 95 °C for 5 min to lyse cells and then placed on ice for at least 5 min. Samples and controls were tested with all assays presented in this study.

## 5. Conclusions

The assays described here were validated and shown to be fit-for-purpose. All assays demonstrated high sensitivity and specificity, as well as the ability to detect the pathogen in inoculated plant samples, both symptomatic and asymptomatic. Four different assay variations were validated in this study, giving options to the end-users as to whether they want to simply detect *R. pseudosolanacearum* or differentiate phylotypes I and III, while accommodating two different portable instruments. Assay 1 is a real-time LAMP assay that detects *R. pseudosolanacearum* and can be run with any instrument that can read FAM fluorescence. In this study, Assay 1 was run in the QuantStudio™ 5 Real-Time PCR System for large-scale testing and then validated further in the AmpliFire^®^ Isothermal Fluorometer and Genie^®^ III. Assays 2 and 3 are real-time LAMP assays that differentiate *R. pseudosolanacearum* phylotypes I and III; the phylotype III probe was tagged with HEX and ATTO590 for compatibility with the AmpliFire^®^ Isothermal Fluorometer and Genie^®^ III, respectively. Assay 2 was also run in the CFX Opus Real-Time PCR System for large-scale testing. Assay 4 is a colorimetric LAMP assay that detects *R. pseudosolanacearum* and can be run in a simple heat block or any other simplified device that can maintain a constant 65 °C. Assays 1 and 4 were designed to detect *R. pseudosolanacearum* (phylotypes I and III) but can also be used to differentiate phylotypes I and III following initial detection by running the CP and HP primer sets separately. The assays presented here represent useful diagnostic tools that can be deployed on-site to aid domestic survey and detection efforts. These assays were validated using many *Ralstonia* strains; however, as with any assay, performance of these assays should be continuously monitored, especially regarding specificity, as new strains will arise over time.

## Figures and Tables

**Table 1:** Results for Euphresco samples and controls tested using all LAMP assays.

Sample	Sample Identity	Assay 1 (AmpliFire)	Assay 1 (Genie III)	Assay 2 (AmpliFire)		Assay 3 (Genie III)		Assay 4
				Phylotype I	Phylotype III	Phylotype I	Phylotype III	
1	I (PD7123) in rose stem extract	+	+	+	−	+	−	+
2	I (PD8383) in ginger rhizome extract	+	+	+	−	+	−	+
3	IV (R784) in potato tuber extract	−	−	−	−	−	−	−
4	Surface water	−	−	−	−	-	−	−
5	I (PD8131) and II (PD7106) in surface water	+	+	+	−	+	−	+
6	Potato tuber extract	−	−	−	−	−	−	−
7	III (GBBC3272) in potato tuber extract	+	+	−	+	−	+	+
8	II (PD7421) in rose stem extract	−	−	−	−	−	−	−
9	IV (MAFF301558) in potato tuber extract	−	−	−	−	−	−	−
10	Ginger rhizome extract	−	−	−	−	−	−	−
PC1	*R. pseudosolanacearum* (I, PD7123)	+	+	+	−	+	−	+
PC2	*R. solanacearum* (II, PD7106)	−	−	−	−	−	−	−
PC3	*R. pseudosolanacearum* (III, GBBC3272)	+	+	−	+	−	+	+
PC4	*R. syzygii* subsp. *indonesiensis* (IV, R784)	−	−	−	−	−	−	−

**Table 2:** Oligonucleotide primers and probes developed or used in this study ^a^.

Oligonucleotide	Sequence (5′ → 3′)	Gene Target	Phylotype	Ref.
CP-F3	GAACACCGCCCCAACG	Conserved protein of unknown function	I	This study
CP-B3	GATGAGCAGCGGAAAGGC			
CP-FIP	GGCCCAGATCGTGGAGTCCTGTTTATCGACGATTTCCTGACGGA			
CP-BIP	GCAACCGCTATGCCCACGGTTTGGCAATCTGCAGCACCAG			
CP-LB	CTTCACCGGCTTCAACGCAC			
CP-LB-FAM	/56-FAM/ACGCTGAGGACCCGGATGCGAATGCGGATGCGGATGCCGATTCTTCACCGGCTTCAACGCAC			
HP-F3	GCGCTGCTGGCGAACA	Hypothetical protein	III	This study
HP-B3	GTCGTTGCGTACTGCTCAG			
HP-FIP	GTTCACCCACTCGGCCTGGTTTGATGGCCGATCGCTACC			
HP-BIP	CCGCTCAAGGGTTTGCCGATGTTTCACGCATGTCCATTCCGT			
HP-LF	GGCTTCACCCCGATCCA			
HP-LB	GGGTATCAAGCACATGCTGAG			
HP-LB-FAM	/56-FAM/ACGCTGAGGACCCGGATGCGAATGCGGATGCGGATGCCGATTGGGTATCAAGCACATGCTGAG			
HP-LB-HEX	/5HEX/ACGCTGAGGACCCGGATGCGAATGCGGATGCGGATGCCGATTGGGTATCAAGCACATGCTGAG			
HP-LB-ATTO590	/5ATTO590N/ACGCTGACGCCACTGCCAGACTGACCCAAGCTCGTACCGATTGGGTATCAAGCACATGCTGAG			
Q1-IBFQ	TCGGCATCCGCATCCGCATTCGCATCCGGGTCCTCAGCGT/3IABkFQ/	N/A	N/A	[29,54]; This study
Q2-IBRQ	TCGGTACGAGCTTGGGTCAGTCTGGCAGTGGCGTCAGCGT/3IAbRQSp/	N/A	N/A	This study
Rs16S-F	GCGGGGGTAGCTTGCTAC	16S rRNA	All	[55]
Rs16S-R	GTTATCCCCCACTACAGGGCAC			
Rs16S-P	/56-FAM/TTCAAGCCGAACACCTGCTGCAAG/3BHQ_1/			

^a^ Oligonucleotides used in this study were synthesized by Integrated DNA Technologies (IDT, Coralville, IA, USA).

## Data Availability

The original contributions presented in this study are included in the article/supplementary material. Further inquiries can be directed to the corresponding author.

## Supplementary Materials

The supporting information can be downloaded at https://www.mdpi.com/article/10.3390/ijms27208976/s1.
