# Supplementary material for: Genome-Informed Loop-Mediated Isothermal Amplification (LAMP) Assays for Detection and Differentiation of Ralstonia pseudosolanacearum Phylotypes I and III
Source: Int J Mol Sci. 2026 Oct 10;27(20):8976. doi: 10.3390/ijms27208976 (PMC13704481; doi:10.3390/ijms27208976)
Supplement: Supplementary file 1 [file ijms-27-08976-s001.zip › ijms-4605288-supplementary.pdf]

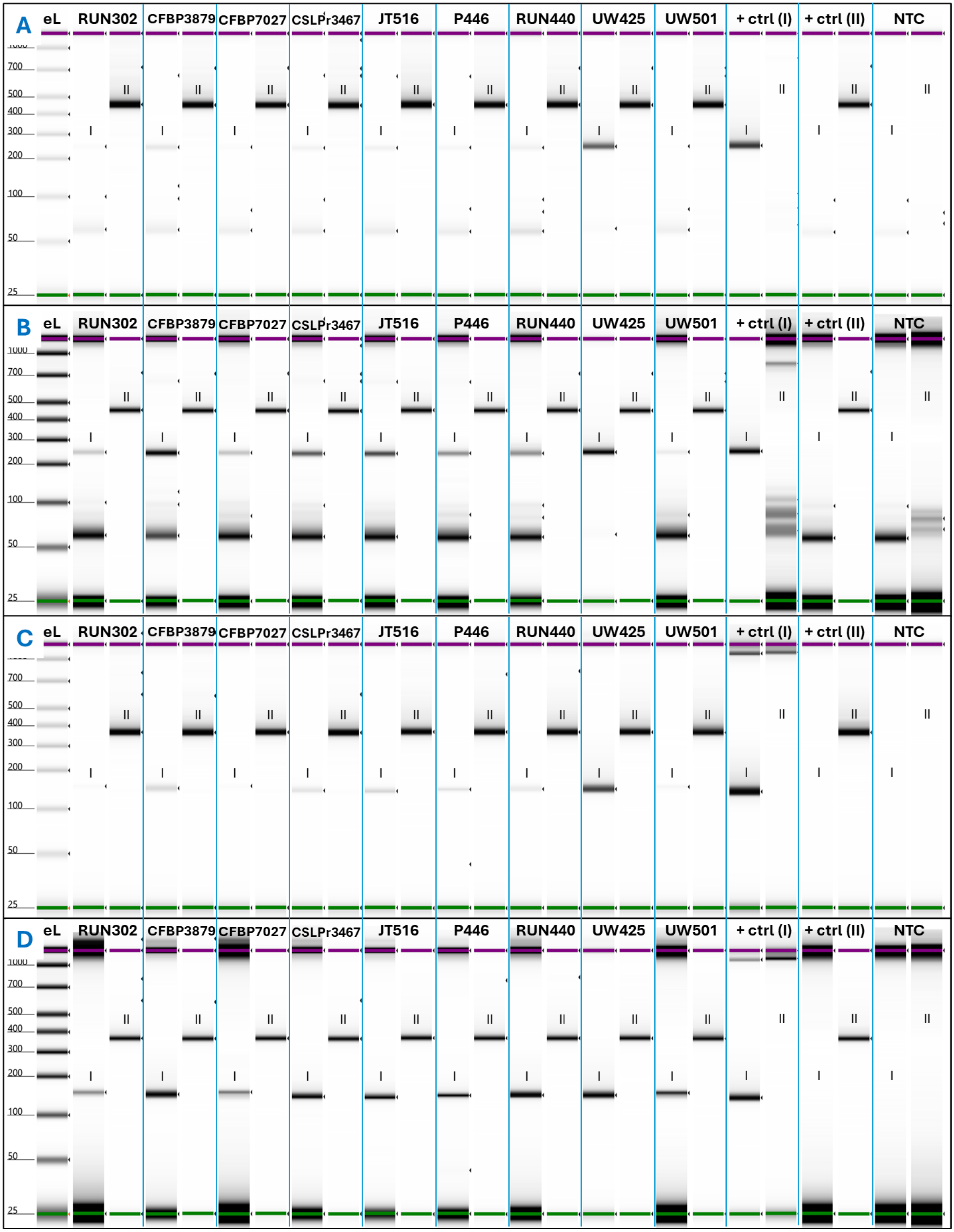
A
B
C
D
eL
RUN302
CFBP3879
CFBP7027
CSLPr3467
JT516
P446
RUN440
UW425
UW501
+ ctrl (I)
+ ctrl (II)
NTC
1000
700
500
400
300
200
100
50
25
I
II

Figure S1. Gel image showing amplicons generated by conventional PCR using protocol from Fagan and Prior (2005) and Paudel et al. (2024). Nine *Ralstonia solanacearum* (phylotype II) strains testing positive by the *R. pseudosolanacearum* LAMP assay were subjected to cPCR amplification according to modified Paudel et al. (2024) (**A** and **B**) and Fagan and Prior (2005) (**C** and **D**) protocols, using only the phylotype I and II primer sets in single-plex format. Amplicons were analyzed using the 4200 TapeStation System, D1000 ScreenTape, and D1000 Sample Buffer, according to the manufacturer's instructions. **A) and C)** Default image; **B) and D)** Scaled to sample for improved viewing of faint bands. **eL** = electronic ladder; **+ ctrl (I)** = confirmed pure culture of 24092601-01 (phylotype I); **+ ctrl (II)** = confirmed pure culture of Molk2 (phylotype IIB). Data shows that all strains were phylotype II, as seen by the bright bands produced with the phylotype II-specific primer sets (A and B = 478 bp; C and D = 372 bp); however, these samples also contained phylotype I, as seen by the fainter bands produced by the phylotype I-specific primer sets (A and B = 251 bp; C and D = 144 bp). Sample UW425 produced the brightest bands with the phylotype I-specific primer sets and also had the fastest positive results by the LAMP assay. Phylotype I and II positive controls produced a band only for their respective phylotypes; no bands were produced with the NTC. The presence of phylotype I was confirmed by three independent phylotype I-specific assays (two cPCR and LAMP) that target different unique markers and produced positive results.

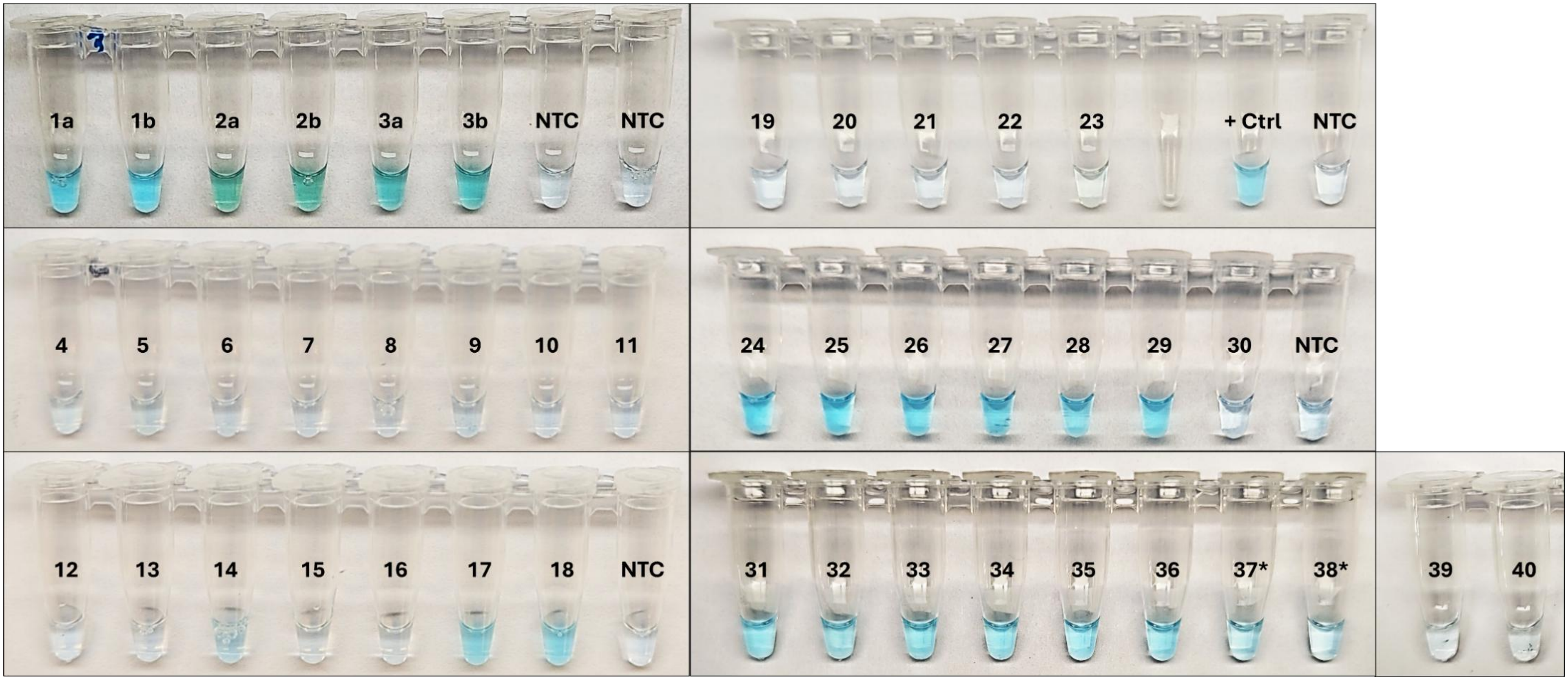

Figure S2. Colorimetric LAMP assay (Assay 4) results for inoculated plant samples. Different host plants were inoculated with different phylotype I and III isolates across two laboratories (PPCDL and ARS). Plants were sampled according to a field-applicable protocol and then tested with the colorimetric LAMP assay (Assay 4). **1-30)** Plant samples from PPCDL; **31-40)** Plant samples from ARS. 1)Potato-1 with UW685 (III), symptomatic; 2) Geranium-1 with UW685 (III), symptomatic; 3) Geranium-2 with UW685 (III), symptomatic; 4) Geranium-1 with 202402-02 (I), asymptomatic; 5) Geranium-2 with 202402-02 (I), asymptomatic; 6) Ginger-1 with 202402-02 (I), asymptomatic; 7) Ginger-2 with 202402-02 (I), asymptomatic; 8) Ginger-1 with UW685 (III), asymptomatic; 9) Ginger-2 with UW685 (III), asymptomatic; 10) Pepper-1 with 202402-02 (I), asymptomatic; 11) Pepper-1 with UW685 (III), asymptomatic; 12) Potato-1 with 202402-02 (I), asymptomatic; 13) Potato-2 with 202402-02 (I), asymptomatic; 14) Potato-2 with UW685 (III), asymptomatic; 15) Tomato-1 with 202402-02 (I), asymptomatic; 16) Tomato-2 with 202402-02 (I), asymptomatic; 17) Tomato-1 with UW685 (III), asymptomatic; 18) Tomato-2 with UW685 (III), asymptomatic; 19) Geranium, healthy; 20) Ginger, healthy; 21) Pepper, healthy; 22) Potato, healthy; 23) Tomato, healthy; 24) Rose-2-1a ('Papa Meilland®') with PD7123 (I-33), asymptomatic; 25) Rose-2-1b ('Papa Meilland®') with PD7123 (I-33), asymptomatic; 26) Rose-2-1c ('Papa Meilland®') with PD7123 (I-33), asymptomatic; 27) Rose-2-7b ('Papa Meilland®') with PD7123 (I-33), asymptomatic; 28) Rose-2-7c ('Papa Meilland®') with PD7123 (I-33), asymptomatic; 29) Rose-3 ('Pink Double Knock Out®') with UW764 (I-18), asymptomatic; 30) Rose-2 ('Papa Meilland®'), healthy; 31) Tomato-1 with Rs121; 32) Tomato-8 with Rs121; 33) Tomato-1 with Pss262; 34) Tomato-8 with Pss262; 35) Tomato-1 with GMI1000; 36) Tomato-8 with GMI1000; 37) Tomato-1 with RUN302; 38) Tomato-8 with RUN302; 39) Tomato-1, healthy; 40) Tomato-4, healthy. NTC – no-template control. Labels "a" and "b" denote different samples taken from the same plant/pot. All symptomatic tissues yielded positive results. Rose samples were processed by a different field extraction protocol, as they were asymptomatic and showed inhibition. Several asymptomatic plants also yielded positive results; positive results were confirmed by 16S real-time PCR; negative LAMP results were also negative by PCR. Healthy (mock-inoculated) plants produced negative results. *RUN302-infected tomatoes tested positive by the LAMP assay, but the RUN302 culture was found to be contaminated with an unknown phylotype I strain.

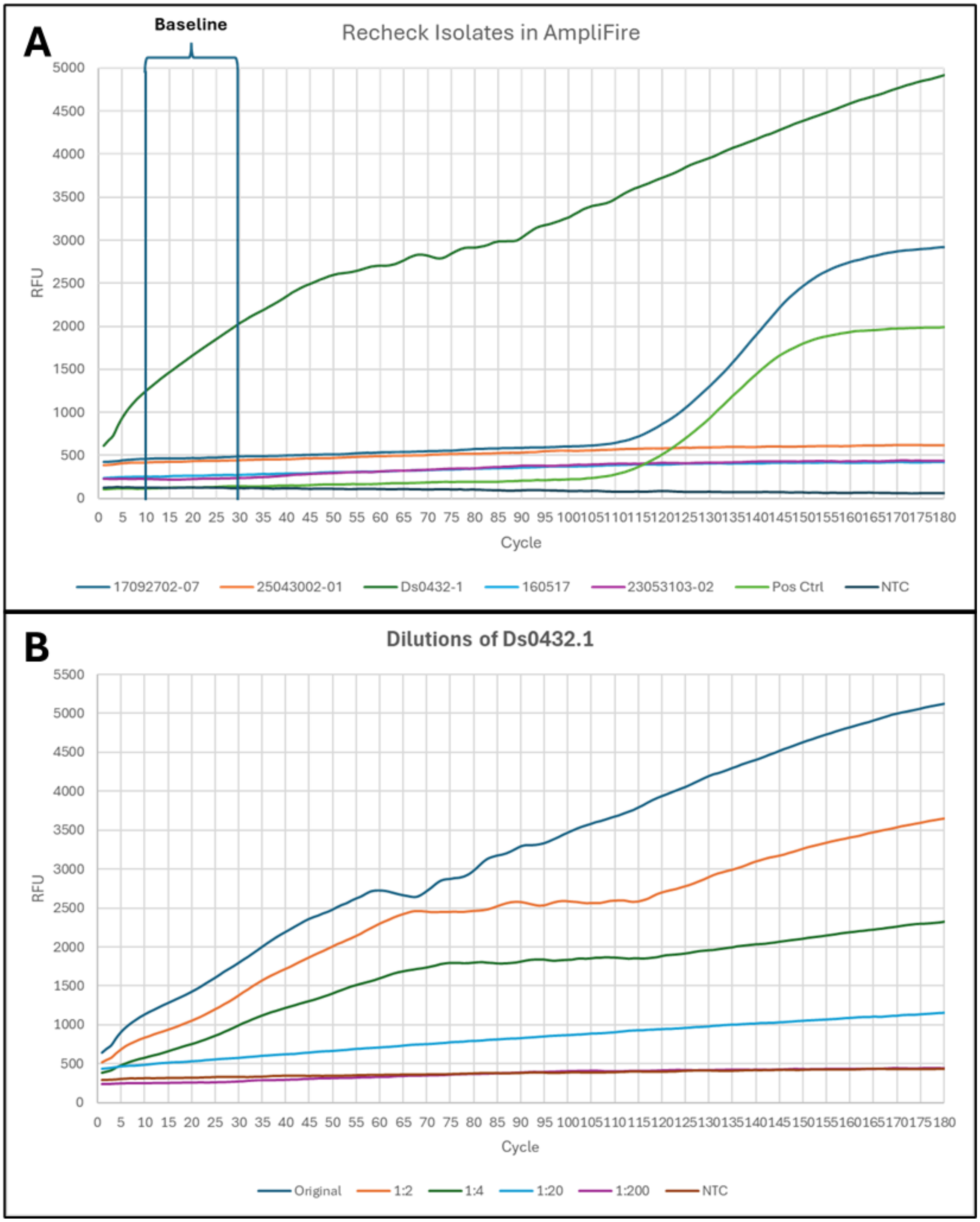

Figure S3. Results of specificity testing using the AmpliFire® Isothermal Fluorometer. **A)** Initial test showing a linear-like amplification curve for *Dickeya solani* Ds0432.1 that starts immediately and was determined positive by the instrument; normal positive amplification curves are shown for comparison. This phenomenon was observed in other instruments, including the Bio-Rad OPUS and Genie® III. This sample was found to contain an unknown amount of DMSO, which was used as a cryoprotectant. **B)** The sample was diluted and the curves reduced correspondingly. At a 1:20 dilution, which corresponds to ~50 ng per reaction, the AmpliFire returned a result assessment of negative. Results demonstrate that the erroneous amplification was due to the presence of DMSO in the sample, which was denaturing the probe-quencher complex and releasing positive fluorescent signal.

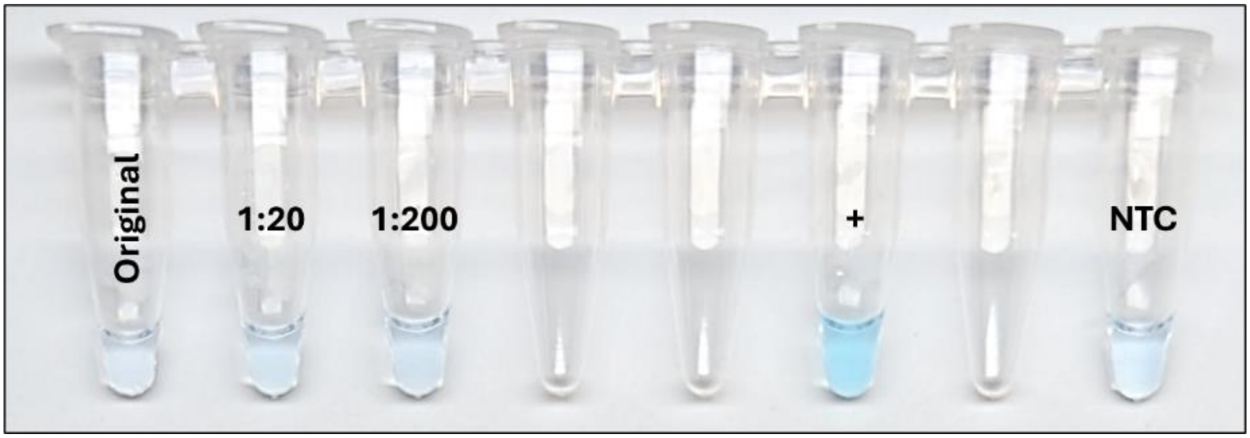

Figure S4. Colorimetric LAMP assay results for *Dickeya solani* Ds0432.1. The original Ds0432.1 sample and 1:20 and 1:200 dilutions were tested with the colorimetric LAMP assay. Results showed that the Ds0432.1 sample and its dilutions produced negative results; no observed color change relative to the no-template control (NTC). The colorimetric LAMP assay contains a dye that changes the reaction color based on the production of dual-stranded DNA during amplification. These negative results confirm that the erroneous signal produced with the real-time assays was due to denaturation of the probe-quencher complex, and not actual amplification of target DNA.

A) B)

Figure S5. AmpliFire® Isothermal Fluorometer reaction QR codes. **A)** Assay 1 – real-time LAMP for detection of *Ralstonia pseudosolanacearum*. **B)** Assay 2 - real-time LAMP for detection and differentiation of *Ralstonia pseudosolanacearum* phylotypes I and III. QR codes are scanned by the AmpliFire® Isothermal Fluorometer and automatically load the reaction conditions and analysis parameters. Result outputs are binary (+/-).

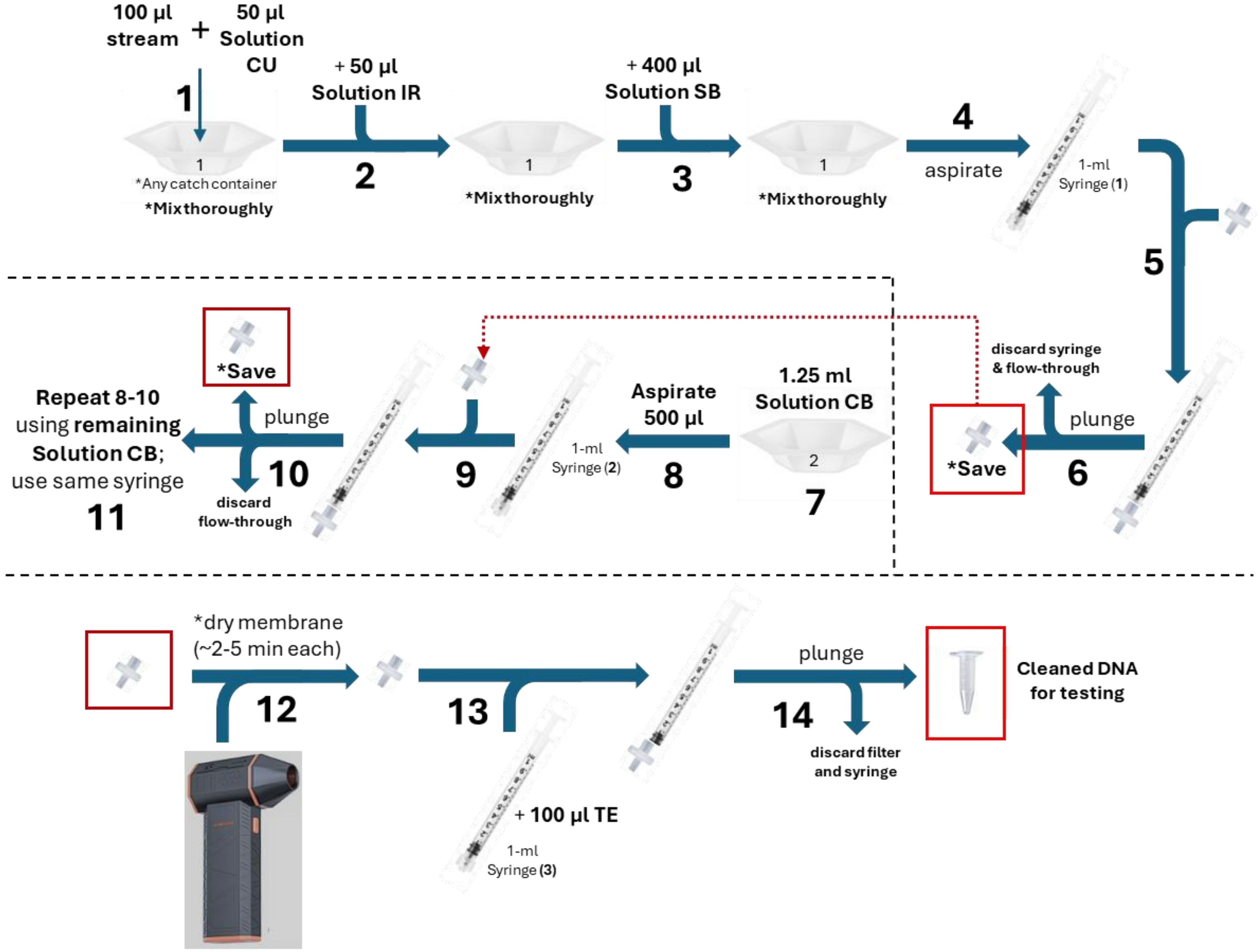

Figure S6. Graphical schematic illustrating the workflow for cleaning DNA from streamed samples in the field.

Table S1. 16S rRNA real-time PCR results for serial dilutions of *R. pseudosolanacearum* phylotype I strain 202402-02 and phylotype II strains UW386 and UW472 DNA.

				24092601-01 (Phylotype I)				UW386 (Phylotype III)				UW472 (Phylotype III)			
**Dilution**	**~GEC/µl**	**~GEC/rxn**	**N**	**Mean**	**Std Dev**	**CV (%)**	**% Positive**	**Mean**	**Std Dev**	**CV (%)**	**% Positive**	**Mean**	**Std Dev**	**CV (%)**	**% Positive**
0	165,895	829,475	3	16.35	0.20	1.22%	100.00%	17.13	0.13	0.75%	100.00%	16.08	0.04	0.23%	100.00%
1	16,590	82,948	3	19.67	0.32	1.63%	100.00%	20.30	0.10	0.49%	100.00%	19.72	0.15	0.76%	100.00%
2	1,659	8,295	3	23.24	0.25	1.10%	100.00%	23.37	0.12	0.52%	100.00%	23.26	0.26	1.11%	100.00%
3	166	829	3	27.12	0.11	0.40%	100.00%	27.05	0.10	0.35%	100.00%	27.07	0.16	0.57%	100.00%
3.5	52	262	3	29.12	0.15	0.50%	100.00%	28.95	0.17	0.59%	100.00%	29.16	0.21	0.74%	100.00%
4	17	83	3	31.40	0.07	0.23%	100.00%	31.26	0.21	0.67%	100.00%	31.85	0.17	0.53%	100.00%
4.5	5	26	3	33.68	0.23	0.69%	100.00%	33.38	0.24	0.71%	100.00%	34.66	0.47	1.36%	100.00%
5	2	8	3	36.54	0.50	1.37%	100.00%	36.30	0.44	1.22%	100.00%	36.76	0.56	1.53%	100.00%

GEC = genome equivalent copies; calculated using the following formula: $\text{number of copies (molecules)} = \dfrac{\mathbf{X}\,\text{ng} * 6.0221\times10^{23}\frac{\text{molecules}}{\text{mole}}}{\left(\mathbf{N} * 660\frac{\text{g}}{\text{mole}*\text{bp}}\right) * 1\times10^{9}\frac{\text{ng}}{\text{g}}}$

N = number of replicates

Rxn = reaction

Mean = average cycle threshold (C_t) value

Std Dev = standard deviation

CV = coefficient of variation (Std Dev / Mean)

Table S2. Bacterial strains used in this study and their corresponding LAMP assay results.[a]

Organism	Strain	Alternate ID	Phylotype	Seq	Host	Host (Common)	Location	Source[b]	Assay 1 (FAM)	Assay 2	
										FAM (I)	HEX (III)
Ralstonia pseudosolanacearum	1	A3313; K0101	I	18	*Solanum lycopersicum*	Tomato	Australia	M. Arif	+	+	-
Ralstonia pseudosolanacearum	171	A3315; K0103	I	-	*Solanum melongena*	Eggplant	Australia	M. Arif	+	+	-
Ralstonia pseudosolanacearum	190	A3318; K0106	I	18	*Xanthium pungens*	Common cocklebur	Australia	M. Arif	+	+	-
Ralstonia pseudosolanacearum	234	A3319; K0107	I	18	*Pinguicula villosa*	Hairy butterwort	Australia	M. Arif	+	+	-
Ralstonia pseudosolanacearum	1017	A3316; K0104	I	18	*Solanum nigrum*	Black nightshade	Australia	M. Arif	+	+	-
Ralstonia pseudosolanacearum	1105B	A3536; K0142	I	18	*Arachis hypogaea*	Peanut	Indonesia	M. Arif	+	+	-
Ralstonia pseudosolanacearum	1605WS	A3527; K0133	I	-	*Arachis hypogaea*	Peanut	Indonesia	M. Arif	+	+	-
Ralstonia pseudosolanacearum	19127	S-6; A6378	I	-	*Casuarina equisetifolia*	Ironwood	Guam	M. Arif	+	+	-
Ralstonia pseudosolanacearum	19147	S-1; A6398	I	-	*Casuarina equisetifolia*	Ironwood	Guam	M. Arif	+	+	-
Ralstonia pseudosolanacearum	19170	S-5; A6116	I	46	*Casuarina equisetifolia*	Ironwood	Guam	M. Arif	+	+	-
Ralstonia pseudosolanacearum	19202	S-2; A6396	I	-	*Casuarina equisetifolia*	Ironwood	Guam	M. Arif	+	+	-
Ralstonia pseudosolanacearum	19227	S-4; A6405	I	-	*Casuarina equisetifolia*	Ironwood	Guam	M. Arif	+	+	-
Ralstonia pseudosolanacearum	A12	A3772; K0144	I	56	*Momordica charantia*	Bitter melon	Philippines	M. Arif	+	+	-
Ralstonia pseudosolanacearum	A3290	A476-1A; K0097	I	-	*Capsicum annuum*	Pepper	USA, HI	M. Arif	+	+	-
Ralstonia pseudosolanacearum	A3344	A476-1B; K0108	I	-	*Capsicum annuum*	Bell Pepper	USA, HI	M. Arif	+	+	-
Ralstonia pseudosolanacearum	A4679	K0023	I	-	*Zingiber officinale*	Kahili Ginger	USA, HI	M. Arif	+	+	-
Ralstonia pseudosolanacearum	A4736	K0690	I	-	*Solanum lycopersicum*	Tomato	USA, HI	M. Arif	+	+	-
Ralstonia pseudosolanacearum	BB	A5370; K0003	I	15	*Solanum lycopersicum*	Tomato	USA, HI	M. Arif	+	+	-
Ralstonia pseudosolanacearum	BI-13	A5199; K0705	I	30	*Zingiber officinale*	Ginger	USA, HI	M. Arif	+	+	-
Ralstonia pseudosolanacearum	BIOTE1699	A4131; K0165	I	15	*Musa* sp.	Banana	Philippines	M. Arif	+	+	-
Ralstonia pseudosolanacearum	BN-1	A3343; K0767	I	14	*Solanum lycopersicum*	Tomato	USA, HI	M. Arif	+	+	-
Ralstonia pseudosolanacearum	Bv4-90.5	A3587; K0143	I	-	*Ipomoea batatas*	Sweet Potato	China	M. Arif	+	+	-
Ralstonia pseudosolanacearum	C2	A5498; K0758	I	47	*Capsicum annuum*	Chili pepper	India	M. Arif	+	+	-
Ralstonia pseudosolanacearum	C2	A3777; K0146	I	56	*Arachis hypogaea*	Peanut	Philippines	M. Arif	+	+	-
Ralstonia pseudosolanacearum	EB 2	A5491; K0751	I	47	*Solanum melongena*	Eggplant	India	M. Arif	+	+	-
Ralstonia pseudosolanacearum	FJ47	-	I	-	*Solanum lycopersicum*	Tomato	China	Q. Huang	+	+	-
Ralstonia pseudosolanacearum	GMI1000	JS753; RUN54	I	18	*Solanum lycopersicum*	Tomato	French Guyana	M. Arif	+	+	-
Ralstonia pseudosolanacearum	GW-1	A4515; K0022	I	30	*Zingiber officinale*	Ginger	USA, HI	M. Arif	+	+	-
Ralstonia pseudosolanacearum	GX53	-	I	-	*Capsicum annuum*	Pepper	China	Q. Huang	+	+	-
Ralstonia pseudosolanacearum	HB512	-	I	-	*Solanum lycopersicum*	Tomato	China	Q. Huang	+	+	-
Ralstonia pseudosolanacearum	JS526	-	I	-	*Solanum lycopersicum*	Tomato	China	Q. Huang	+	+	-
Ralstonia pseudosolanacearum	JS2082	-	I	-	*Solanum melongena*	Eggplant	China	Q. Huang	+	+	-

Ralstonia pseudosolanacearum	MT1	A3784; K0150; K0674	I	13	*Musa textilis*	Banana (Abacá)	Philippines	M. Arif	+	+	-
Ralstonia pseudosolanacearum	NCPPB0790	UW4; S111; K217; UCD164	I	-	*Physalis angulata*	Gooseberry	Costa Rica	T. Lowe-Power	+	+	-
Ralstonia pseudosolanacearum	PD7123	UW770; Rs4001; UCD396	I	33	*Rosa* sp.	Rose	Netherlands	T. Lowe-Power	+	+	-
Ralstonia pseudosolanacearum	PD8131	-	I	33	-	Surface water	Netherlands	M.J.C. Pel	+	+	-
Ralstonia pseudosolanacearum	PO-7	A5192; K0024	I	-	*Zingiber officinale*	Ginger	USA, HI	M. Arif	+	+	-
Ralstonia pseudosolanacearum	Pss4	-	I	15	*Solanum lycopersicum*	Tomato	Taiwan	Q. Huang	+	+	-
Ralstonia pseudosolanacearum	Pss97	-	I	34	*Solanum lycopersicum*	Tomato	Taiwan	Q. Huang	+	+	-
Ralstonia pseudosolanacearum	Pss106	-	I	34	*Capsicum annuum*	Pepper	Taiwan	Q. Huang	+	+	-
Ralstonia pseudosolanacearum	Pss114	-	I	-	*Capsicum annuum*	Pepper	Taiwan	Q. Huang	+	+	-
Ralstonia pseudosolanacearum	Pss1283	-	I	-	*Ipomoea aquatica*	Water spinach	Taiwan	Q. Huang	+	+	-
Ralstonia pseudosolanacearum	Pss185	-	I	-	*Solanum melongena*	Eggplant	Taiwan	Q. Huang	+	+	-
Ralstonia pseudosolanacearum	Pss191	-	I	15	*Solanum lycopersicum*	Tomato	Taiwan	Q. Huang	+	+	-
Ralstonia pseudosolanacearum	Pss1655	-	I	-	*Ipomoea batatas*	Sweet Potato	Taiwan	Q. Huang	+	+	-
Ralstonia pseudosolanacearum	Pss262	-	I	-	*Solanum tuberosum*	Potato	Taiwan	Q. Huang	+	+	-
Ralstonia pseudosolanacearum	Rs121	-	I	13	*Capsicum annuum*	Pepper	USA, FL	Q. Huang	+	+	-
Ralstonia pseudosolanacearum	S25	S-3; A6377	I	-	*Casuarina equisetifolia*	Ironwood	Guam	M. Arif	+	+	-
Ralstonia pseudosolanacearum	SM714	UCD497; COM-C-01	I	48	*Capsicum annuum*	Pepper	Bangladesh	T. Lowe-Power	+	+	-
Ralstonia pseudosolanacearum	SM1850	UCD520; BIR-E-06	I	48	*Solanum melongena*	Eggplant	Nepal	T. Lowe-Power	+	+	-
Ralstonia pseudosolanacearum	SQ21	A3780; K0149	I	56	*Cucurbita* sp.	Squash	Philippines	M. Arif	+	+	-
Ralstonia pseudosolanacearum	T6	A3776; K0145	I	-	*Solanum lycopersicum*	Tomato	Philippines	M. Arif	+	+	-
Ralstonia pseudosolanacearum	TM2 90.5	A3588; K0768	I	30	*Solanum lycopersicum*	Tomato	China	M. Arif	+	+	-
Ralstonia pseudosolanacearum	TZ3	-	I	31	*Capsicum annuum*	Pepper	Tanzania	T. Lowe-Power	+	+	-
Ralstonia pseudosolanacearum	UW6	A4492; K0159; K0147	I	14	*Heliconia* sp.	-	Costa Rica	M. Arif	+	+	-
Ralstonia pseudosolanacearum	UW8	A3461; K0123	I	-	*Chromolaena odorata*	Siam weed	Costa Rica	M. Arif	+	+	-
Ralstonia pseudosolanacearum	UW27	A3457; K0119	I	15	*Nicotiana* sp.	Tobacco	USA, FL	M. Arif	+	+	-
Ralstonia pseudosolanacearum	UW119	A3460; K0122	I	14	*Solanum tuberosum*	Potato	Costa Rica	M. Arif	+	+	-
Ralstonia pseudosolanacearum	UW141	A3466; K0128	I	16	*Zingiber officinale*	Ginger	Australia	M. Arif	+	+	-
Ralstonia pseudosolanacearum	UW151		I	16	*Zingiber officinale*	Ginger	Australia	Q. Huang	+	+	-
Ralstonia pseudosolanacearum	UW152	A3456; K0118; S241	I	18	*Solanum tuberosum*	Potato	Australia	M. Arif	+	+	-
Ralstonia pseudosolanacearum	UW255	A3446; K0110	I	-	*Capsicum annuum*	Pepper	Costa Rica	M. Arif	+	+	-
Ralstonia pseudosolanacearum	UW329	A5660	I	15	*Nicotiana tabacum*	Tobacco	Taiwan	M. Arif	+	+	-
Ralstonia pseudosolanacearum	UW338	UCD264	I	-	*Psophocarpus tetragonolobus*	Winged bean	Malaysia	T. Lowe-Power	+	+	-
Ralstonia pseudosolanacearum	UW355	A5661	I	15	*Ipomoea batatas*	Sweet Potato	China	M. Arif	+	+	-
Ralstonia pseudosolanacearum	UW359	A3452; K0114	I	16	*Zingiber officinale*	Ginger	China	M. Arif	+	+	-

Ralstonia pseudosolanacearum	UW360	A3464; K0126	I	48	*Morus* sp.	Mulberry	China	M. Arif	+	+	-
Ralstonia pseudosolanacearum	UW361	A5662; CIP277; JT661; R292; RUN91	I	12	*Morus alba*	Mulberry	China	M. Arif	+	+	-
Ralstonia pseudosolanacearum	UW373	A3462; R288; CFBP6442; RUN90	I	48	*Morus alba*	Mulberry	China	M. Arif	+	+	-
Ralstonia pseudosolanacearum	UW378	A3448; K0111	I	-	*Olea europaea*	Olive	China	M. Arif	+	+	-
Ralstonia pseudosolanacearum	UW380	A3467; K0129	I	-	*Olea europaea*	Olive	China	M. Arif	+	+	-
Ralstonia pseudosolanacearum	UW672	RUN215; CFBP7058; CMR134; UCD360	I	13	*Solanum scabrum*	Garden huckleberry	Cameroon	T. Lowe-Power	+	+	-
Ralstonia pseudosolanacearum	UW682	A5664	I	-	*Arachis hypogaea*	Peanut	China	C. Allen	+	+	-
Ralstonia pseudosolanacearum	UW762	EP1	I	34	*Solanum melongena*	Eggplant	China	C. Allen	+	+	-
Ralstonia pseudosolanacearum	UW763	UCD392	I	14	*Solanum lycopersicum*	Tomato	Senegal	C. Allen	+	+	-
Ralstonia pseudosolanacearum	UW764	UCD393	I	18	*Rosa* sp.	Rose	Ukraine	T. Lowe-Power	+	+	-
Ralstonia pseudosolanacearum	UW784	RUN155, PSS366	I	15	*Solanum lycopersicum*	Tomato	Taiwan	C. Allen	+	+	-
Ralstonia pseudosolanacearum	UW801	B18, UCD424	I	-	*Vernonia amygdalina*	Bitter leaf	Benin	T. Lowe-Power	+	+	-
Ralstonia pseudosolanacearum	UW810	T24; UCD433	I	17	*Capsicum annuum*	Pepper	Togo	T. Lowe-Power	+	+	-
Ralstonia pseudosolanacearum	WP17	A3779; K0148	I	18	*Solanum tuberosum*	White Potato	Philippines	M. Arif	+	+	-
Ralstonia pseudosolanacearum	ZO 36	A3786; K0021	I	-	*Zingiber officinale*	Ginger	Philippines	M. Arif	+	+	-
Ralstonia pseudosolanacearum	CFBP3059	-	III	23	*Solanum melongena*	Eggplant	Burkina Faso	M. Arif	+	-	+
Ralstonia pseudosolanacearum	CFBP6942	UCD789; UW664; CMR32	III	29	*Solanum scabrum*	Garden huckleberry	Cameroon	M. Arif	+	-	+
Ralstonia pseudosolanacearum	CIP296	UW472, A5676	III	43	*Solanum tuberosum*	Potato	Nigeria	M. Arif	+	-	+
Ralstonia pseudosolanacearum	J25	RUN56; KE056	III	20	*Solanum tuberosum*	Potato	Kenya	M. Arif	+	-	+
Ralstonia pseudosolanacearum	JT525	RUN60	III	19	*Pelargonium* sp.	Geranium	Réunion	M. Arif	+	-	+
Ralstonia pseudosolanacearum	LMG 9673^T	UQRS 461; ICMP 769; NCPPB 1029; RUN77	III	19	*Pelargonium asperum*	Geranium	Réunion	M. Arif	+	-	+
Ralstonia pseudosolanacearum	NCPPB332	-	III	22	*Solanum tuberosum*	Potato	Zimbabwe	M. Arif	+	-	+
Ralstonia pseudosolanacearum	UW386	UCD284	III	23	-	Soil	Nigeria	C. Allen	+	-	+
Ralstonia pseudosolanacearum	UW685	RUN362; DGBBC1138; UCD366	III	44	*Solanum tuberosum*	Potato	Guinea	C. Allen	+	-	+
Ralstonia pseudosolanacearum	UW686	UCD792; DGBBC1227	III	42	*Solanum tuberosum*	Potato	Guinea	T. Lowe-Power	+	-	+
Ralstonia pseudosolanacearum	UW691	RUN479, NCPPB1018, JS950	III	21	*Solanum tuberosum*	Potato	Angola	C. Allen	+	-	+
Ralstonia pseudosolanacearum	UW773	RUN2279	III	60	*Solanum tuberosum*	Potato	Madagascar	C. Allen	+	-	+
Ralstonia pseudosolanacearum	UW774	RUN2474	III	19	*Solanum tuberosum*	Potato	Madagascar	C. Allen	+	-	+
Ralstonia pseudosolanacearum	UW777	RUN2706; UCD402	III	19	*Solanum tuberosum*	Potato	Madagascar	T. Lowe-Power	+	-	+
Ralstonia pseudosolanacearum	UW780	RUN3235; UCD404	III	60	*Solanum tuberosum*	Potato	Madagascar	T. Lowe-Power	+	-	+

Ralstonia solanacearum	Rs126	-	II	-	*Pelargonium* sp.	Geranium	USA, FL	Q. Huang	-	-	-
Ralstonia solanacearum	UHM080	-	II	-	*Solanum tuberosum*	Potato	Brazil	M. Arif	-	-	-
Ralstonia solanacearum	UW84	A5658	II	-	*Solanum lycopersicum*	Tomato	Canada	T. Lowe-Power	-	-	-
Ralstonia solanacearum	UW457	A5666	II	-	-	Soil	Peru	T. Lowe-Power	-	-	-
Ralstonia solanacearum	38606	A4380; K0168	IIA	6	*Musa* sp.	Banana	India	M. Arif	-	-	-
Ralstonia solanacearum	CFBP2957	UW635; UCD345	IIA	36	*Solanum lycopersicum*	Tomato	Martinique	T. Lowe-Power	-	-	-
Ralstonia solanacearum	CFBP2958	UW631; RUN028; GT4; UCD344	IIA	39	*Solanum lycopersicum*	Tomato	Guadeloupe	T. Lowe-Power	-	-	-
Ralstonia solanacearum	DP6	A5281; K0741	IIA	6	*Zingiber officinale*	Ginger	USA, HI	M. Arif	-	-	-
Ralstonia solanacearum	KV2.2	A3908	IIA	6	*Heliconia* sp.	Heliconia	USA, HI	M. Arif	-	-	-
Ralstonia solanacearum	P597	-	IIA	38	*Solanum lycopersicum*	Tomato	USA, FL	Q. Huang	-	-	-
Ralstonia solanacearum	UW20	A4495; K0172	IIA	6	*Musa* sp.	Banana	Venezuela	C. Allen	-	-	-
Ralstonia solanacearum	UW90	A3468	IIA	50	*Nicotiana* sp.	Tobacco	Brazil	T. Lowe-Power	-	-	-
Ralstonia solanacearum	UW594	UCD726	IIA	6	*Musa* sp.	Banana	Guatemala	T. Lowe-Power	-	-	-
Ralstonia solanacearum	UW688	RUN448; T1-UY; UCD369	IIA	50	*Solanum lycopersicum*	Tomato	Uruguay	T. Lowe-Power	-	-	-
Ralstonia solanacearum	UW748	Grenada 9-1; UCD383	IIA	6	*Musa* sp.	Banana (Bluggoe)	West Indies	C. Allen	-	-	-
Ralstonia solanacearum	CFBP3879	RUN628; CFBP1414	IIB	2	*Solanum tuberosum*	Potato	Colombia	Q. Huang	+	+	-
Ralstonia solanacearum	CFBP7027	RUN141; CMR24	IIB	1	*Solanum tuberosum*	Potato	Cameroon	Q. Huang	+	+	-
Ralstonia solanacearum	CFBP7029	RUN147; CMR34	IIB	1	*Solanum tuberosum*	Potato	Cameroon	Q. Huang	-	-	-
Ralstonia solanacearum	CFBP8695	K2	IIB	1	*Solanum tuberosum*	Potato	Iran	M. Arif	-	-	-
Ralstonia solanacearum	CFBP8697	Sh16	IIB	25	*Solanum tuberosum*	Potato	Iran	M. Arif	-	-	-
Ralstonia solanacearum	CSLPr1328	-	IIB	1	*Solanum tuberosum*	Potato	Egypt	Q. Huang	-	-	-
Ralstonia solanacearum	CSLPr3467	P.6019	IIB	1	*Solanum tuberosum*	Potato	UK	Q. Huang	+	+	-
Ralstonia solanacearum	CSLPr3468	P.6018	IIB	1	*Solanum lycopersicum*	Tomato	UK	Q. Huang	-	-	-
Ralstonia solanacearum	IVIA1602.1	-	IIB	-	*Solanum tuberosum*	Potato	Spain	Q. Huang	-	-	-
Ralstonia solanacearum	JT516	RUN160	IIB	1	*Solanum tuberosum*	Potato	Réunion	Q. Huang	+	+	-
Ralstonia solanacearum	MolK2	R633; GMI11961; RUN74	IIB	3	*Musa* sp.	Banana	Phillippines	M. Arif	-	-	-
Ralstonia solanacearum	NCPPB1584	-	IIB	1	*Solanum tuberosum*	Potato	Cyprus	Q. Huang	-	-	-
Ralstonia solanacearum	NCPPB2505	-	IIB	1	*Solanum tuberosum*	Potato	Sweden	Q. Huang	-	-	-
Ralstonia solanacearum	P446	-	IIB	4	*Epipremnum aureum*	Pothos	USA, FL	Q. Huang	+	+	-
Ralstonia solanacearum	P618	RUN3	IIB	4	*Anthurium* sp.	Anthurium	Martinique	T. Lowe-Power	-	-	-
Ralstonia solanacearum	P868	-	IIB	4	*Musa acuminata*	Banana	Suriname	T. Lowe-Power	-	-	-
Ralstonia solanacearum	PS-13	A4615	IIB	1	*Pelargonium* sp.	Geranium	Guatemala	M. Arif	-	-	-
Ralstonia solanacearum	PS-31	A4622	IIB	1	*Pelargonium* sp.	Geranium	Guatemala	M. Arif	-	-	-

Ralstonia solanacearum	PS6 (F/D)	A4522; K0009	IIB	3	*Musa* sp.	Banana	Honduras	M. Arif	-	-	-
Ralstonia solanacearum	PS7	A4523; K0177	IIB	3	*Musa* sp.	Banana	Honduras	M. Arif	-	-	-
Ralstonia solanacearum	Pss525	RUN256	IIB	1	*Solanum tuberosum*	Potato	Taiwan	Q. Huang	-	-	-
Ralstonia solanacearum	RUN302	IBSBF1503	IIB	4	*Cucumis sativus*	Cucumber	Brazil	Q. Huang	+	+	-
Ralstonia solanacearum	RUN440	RE	IIB	1	*Solanum tuberosum*	Potato	Uruguay	Q. Huang	+	+	-
Ralstonia solanacearum	RUN651	LNPV24.25	IIB	4	*Solanum lycopersicum*	Tomato	France	Q. Huang	-	-	-
Ralstonia solanacearum	UW9	S147	IIB	3	*Heliconia* sp.	Heliconia	Costa Rica	Q. Huang	-	-	-
Ralstonia solanacearum	UW24	A5689	IIB	1	*Solanum tuberosum*	Potato	Israel	M. Arif	-	-	-
Ralstonia solanacearum	UW37	A5530	IIB	2	*Solanum tuberosum*	Potato	Colombia	M. Arif	-	-	-
Ralstonia solanacearum	UW73	A5532	IIB	1	*Solanum tuberosum*	Potato	Sri Lanka	M. Arif	-	-	-
Ralstonia solanacearum	UW150	S243	IIB	1	*Solanum tuberosum*	Potato	Australia	Q. Huang	-	-	-
Ralstonia solanacearum	UW163	-	IIB	4	*Musa* sp.	Plantain	Peru	T. Lowe-Power	-	-	-
Ralstonia solanacearum	UW220a	-	IIB	1	*Solanum tuberosum*	Potato	India	Q. Huang	-	-	-
Ralstonia solanacearum	UW224	-	IIB	1	*Solanum tuberosum*	Potato	Kenya	Q. Huang	-	-	-
Ralstonia solanacearum	UW344	A5288	IIB	1	*Solanum tuberosum*	Potato	Brazil	M. Arif	-	-	-
Ralstonia solanacearum	UW349	-	IIB	27	*Solanum tuberosum*	Potato	Brazil	Q. Huang	-	-	-
Ralstonia solanacearum	UW365	A5543	IIB	1	*Solanum tuberosum*	Potato	China	M. Arif	-	-	-
Ralstonia solanacearum	UW425	O249	IIB	1	*Solanum tuberosum*	Potato	Australia	Q. Huang	+	+	-
Ralstonia solanacearum	UW449	A5694	IIB	1	*Solanum tuberosum*	Potato	Burundi	M. Arif	-	-	-
Ralstonia solanacearum	UW458	CIP172; A5667	IIB	25	*Solanum tuberosum*	Potato	Peru	C. Allen	-	-	-
Ralstonia solanacearum	UW461	CIP211; A5669	IIB	27	*Solanum tuberosum*	Potato	Brazil	C. Allen	-	-	-
Ralstonia solanacearum	UW473	A5550	IIB	1	*Solanum tuberosum*	Potato	Peru	M. Arif	-	-	-
Ralstonia solanacearum	UW492	CIP302	IIB	1	*Solanum tuberosum*	Potato	Peru	Q. Huang	-	-	-
Ralstonia solanacearum	UW500	A5553	IIB	1	*Solanum tuberosum*	Potato	Indonesia	M. Arif	-	-	-
Ralstonia solanacearum	UW501	CIP181	IIB	1	*Solanum tuberosum*	Potato	Indonesia	Q. Huang	+	+	-
Ralstonia solanacearum	UW550	RUN035; IPO1609	IIB	1	*Solanum tuberosum*	Potato	Netherlands	Q. Huang	-	-	-
Ralstonia solanacearum	UW551	GMI11957; RUN449	IIB	1	*Pelargonium* sp.	Geranium	Kenya	M. Arif	-	-	-
Ralstonia solanacearum	UW552	A5291	IIB	1	*Pelargonium* sp.	Geranium	Guatemala	M. Arif	-	-	-
Ralstonia solanacearum	UW679	RUN299; IBSBF1712; UCD363	IIB	27	*Pelargonium* sp.	Geranium	Brazil	T. Lowe-Power	-	-	-
Ralstonia solanacearum	UW756	RUN981; IBSBF2001; UCD389	IIB	25	*Solanum lycopersicum*	Tomato	Brazil	T. Lowe-Power	-	-	-
Ralstonia solanacearum	K60^T	LMG 2299^T; CFBP2047; UQRS426; NCPPB325; RUN65	IIC	7	*Solanum lycopersicum*	Tomato	USA, NC	M. Arif	-	-	-
Ralstonia solanacearum	P550	-	IIC	7	*Solanum tuberosum*	Potato	USA, FL	Q. Huang	-	-	-

Ralstonia solanacearum	Rs5	A5345; K0004	IIC	7	*Solanum lycopersicum*	Tomato	USA, FL	M. Arif	-	-	-
Ralstonia solanacearum	Rs116	-	IIC	7	*Hydrangea* sp.	Hydrangea	USA, FL	Q. Huang	-	-	-
Ralstonia solanacearum	UW30	A3450; K0005	IIC	7	*Solanum lycopersicum*	Tomato	Trinidad	T. Lowe-Power	-	-	-
Ralstonia solanacearum	UW153	ACH158; S246; Hayward0158; UCD200	IIC	7	*Solanum tuberosum*	Potato	Australia	T. Lowe-Power	-	-	-
Ralstonia solanacearum	UW644	UCD787; ICMP7963	IIC	7	*Solanum tuberosum*	Potato	Kenya	T. Lowe-Power	-	-	-
Ralstonia syzygii	A5487	K0747	IV	11	*Musa* sp.	Banana	Indonesia	M. Arif	-	-	-
Ralstonia syzygii	BD-6	A4612; K0012	IV	-	*Musa* sp.	Banana	Indonesia	M. Arif	-	-	-
Ralstonia syzygii	CFBP6727	UW736; RUN702; UCD377	IV	10	*Solanum tuberosum*	Potato	Indonesia	T. Lowe-Power	-	-	-
Ralstonia syzygii	UW651	MAFF301552; RUN070; JS953; UCD350	IV	8	*Solanum lycopersicum*	Tomato	Japan	T. Lowe-Power	-	-	-
Ralstonia syzygii	UW652	MAFF301558; RUN071; JS934; UCD351	IV	8	*Solanum tuberosum*	Potato	Japan	T. Lowe-Power	-	-	-
Ralstonia syzygii subsp. *celebesensis*	UW742	RUN1348, UQRS465, R230	IV	10	*Musa* sp.	Banana	Indonesia	C. Allen	-	-	-
Ralstonia syzygii subsp. *celebesensis*	UW745	RUN1351, UQRS637	IV	10	*Musa* sp.	Banana	Indonesia	C. Allen	-	-	-
Ralstonia syzygii subsp. *indonesiensis*	LMG 27703^{T}	UQRS 464; DSM 27478; RUN83	IV	10	*Solanum lycopersicum*	Tomato	Indonesia	BCCM LMG	-	-	-
Ralstonia syzygii subsp. *indonesiensis*	UW658	PSI07; UCD354	IV	10	*Solanum lycopersicum*	Tomato	Indonesia	C. Allen	-	-	-
Ralstonia syzygii subsp. *syzygii*	LMG 10661^{T}	R001; DSM 7385; ATCC 49543; NCPPB 3446	IV	9	*Syzygium aromaticum*	Clove	Indonesia	BCCM LMG	-	-	-
Ralstonia syzygii subsp. *syzygii*	UW703	RUN88, R24	IV	9	*Syzygium aromaticum*	Clove	Indonesia	Q. Huang	-	-	-
Ralstonia insidiosa	LMG 21421^{T}	CCUG 46789	-	-	*Homo sapiens*	Human	USA	BCCM LMG	-	-	-
Ralstonia mannitolytica	LMG 6866^{T}	CCUG 38408; CIP 107281;JCM 11284; NCIMB 10805	-	-	*Homo sapiens*	Human	UK	BCCM LMG	-	-	-
Ralstonia pickettii	LMG 5942^{T}	JCM 5969; CCUG 3318; ATCC 27511; ICPB 3981	-	-	*Homo sapiens*	Human	USA	BCCM LMG	-	-	-
Ralstonia thomasii	GP101	-	-	-	-	Soil	-	T. Lowe-Power	-	-	-
Bradyrhizobium elkanii	USDA 76	DSM 11554; LMG 6134; ATCC 49852	-	-	*Glycine max*	Soybean	USA, VA	P. Elia	-	-	-
Clavibacter michiganensis	NRRL B-65397	EF	-	-	*Solanum lycopersicum*	Tomato	USA	NRRL	-	-	-
Clavibacter michiganensis	LMG 7333^{T}	ICMP 2550; ICPB CM177; NCPPB 2979	-	-	*Solanum lycopersicum*	Tomato	Hungary	BCCM LMG	-	-	-
Clavibacter sepedonicus	LMG 2889^{T}	ICMP 2535; ICPB CS101; ATCC 33113; NCPPB 2137	-	-	*Solanum tuberosum*	Potato	Canada	BCCM LMG	-	-	-
Clavibacter sepedonicus	K496	-	-	-	*Solanum tuberosum*	Potato	USA	B. Swingle	-	-	-

Cupriavidus necator	LMG 1199^{T}	335; ATCC 17697; DSM 531; ICPB 3984; NRRL B-4383	-	-	-	Soil	USA	BCCM LMG	-	-	-
Dickeya dadantii subsp. *dieffenbachiae*	646	-	-	-	*Oryza sativa*	Asian cultivated rice	Japan	-	-	-	-
Dickeya dianthicola	160435	-	-	-	Solanum tuberosum	Potato	USA, DE	PPCDL	-	-	-
Dickeya dianthicola	160438	-	-	-	*Solanum tuberosum*	Potato	USA, DE	PPCDL	-	-	-
Dickeya dianthicola	160439	-	-	-	*Solanum tuberosum*	Potato	USA, DE	PPCDL	-	-	-
Dickeya dianthicola	160440	-	-	-	*Solanum tuberosum*	Potato	USA, DE	PPCDL	-	-	-
Dickeya dianthicola	160441	-	-	-	*Solanum tuberosum*	Potato	USA, DE	PPCDL	-	-	-
Dickeya dianthicola	160516	-	-	-	*Solanum tuberosum*	Potato	USA, WV	PPCDL	-	-	-
Dickeya dianthicola	160517	-	-	-	*Solanum tuberosum*	Potato	USA, RI	PPCDL	-	-	-
Dickeya fangzhongdai	19122701-02	-	-	-	*Phalaenopsis* sp.	Moth orchid	USA, CA	PPCDL	-	-	-
Dickeya solani	DS034-1	-	-	-	*Solanum tuberosum*	Potato	-	PPCDL	-	-	-
Dickeya solani	Ds0432-1	LMG 27551; IPO 3295	-	-	*Solanum tuberosum*	Potato	Finland	-	-	-	-
Erwinia aphidicola	17110702-02	-	-	-	*Cucumis melo*	Melon	USA, CA	PPCDL	-	-	-
Erwinia aphidicola	17110702-03	-	-	-	*Cucumis melo*	Melon	USA, CA	PPCDL			
Erwinia aphidicola	18082001-05	-	-	-	*Lupinus* sp.	Lupine	USA, CO	PPCDL	-	-	-
Erwinia persicina	19122705-01	-	-	-	*Achillea millefolium*	Yarrow	USA, CA	PPCDL	-	-	-
Erwinia persicina	19122706-01	-	-	-	*Cucumis melo*	Melon	USA, CA	PPCDL	-	-	-
Erwinia persicina	LA661	-	-	-	*Rubus* sp.	Raspberry	USA, WA	PPCDL	-	-	-
Erwinia persicina	LA659	-	-	-	*Rubus* sp.	Raspberry	USA, WA	PPCDL	-	-	-
Erwinia rhapontici	19122702-01	-	-	-	*Delphinium* sp.	Larkspur	USA, CA	PPCDL	-	-	-
Pantoea ananatis	E	-	-	-	*Oryza sativa*	Rice	USA, AR	J. Leach	-	-	-
Pantoea ananatis	CES-5 (JM-55)	-	-	-	-	Soil	Philippines	S. Miller	-	-	-
Pantoea stewartii	DCop3-07	-	-	-	*Zea mays*	Corn	USA	S. Miller	-	-	-
Pantoea agglomerans	E325	-	-	-	*Malus domestica*	Apple	USA, WA	V. Stockwell	-	-	-
Pantoea agglomerans	23091801-01	-	-	-	Triticum aestivum	Wheat	USA, CO	PPCDL	-	-	-
Pantoea allii	23091801-02	-	-	-	*Triticum aestivum*	Wheat	USA, CO	PPCDL	-	-	-
Pantoea vagans	C9-1	-	-	-	*Malus domestica*	Apple	USA, MI	V. Stockwell	-	-	-
Pectobacterium sp.	160434	-	-	-	*Solanum tuberosum*	Potato	USA, DE	PPCDL			
Pectobacterium sp.	18082801-01	-	-	-	*Aglaonema* sp.	Chinese evergreens	USA, FL	PPCDL	-	-	-
Pectobacterium sp.	23053103-02	-	-	-	*Alocasia* sp.	Elephant ear	USA, VA	PPCDL	-	-	-
Pseudomonas cichorii	DSM 50259^{T}	ATCC 10857; NRRL B-832	-	-	*Cichorium endivia*	Endive (cultivated)	Germany	NRRL	-	-	-
Pseudomonas corrugata	LMG 2172^{T}	ICMP 5819; NCPPB 2445; ATCC 29736; CFBP 2431	-	-	*Solanum lycopersicum*	Tomato	UK	BCCM LMG	-	-	-

Pseudomonas fluorescens	LMG 1794[T]	NCPPB 1964; ICPB 3200; ICMP 3512; DSM 50090	-	-	-	Irrigation water	UK	BCCM LMG	-	-	-
Pseudomonas fluorescens	A506	-	-	-	*Pyrus* sp.	Pear	USA, CA	V. Stockwell	-	-	-
Pseudomonas syringae	JL2583	-	-	-	*Vaccinium* sp.	Blueberry	USA, OR	V. Stockwell	-	-	-
Pseudomonas syringae pv. *syringae*	NCBPP 281[T]	ATCC 19310; NRRL B-1631	-	-	*Syringa vulgaris*	Lilac	UK	NRRL	-	-	-
Pseudomonas syringae pv. *tomato*	NCPPB 996	Pt7; ATCC 10862; NRRL B-882	-	-	*Solanum lycopersicum*	Tomato	Canada	NRRL	-	-	-
Xanthomonas euvesicatoria	LMG 932	NCPPB 2593	-	-	*Capsicum frutescens*	Wild chili pepper	Brazil	BCCM LMG	-	-	-
Xanthomonas fragariae	Balm	-	-	-	*Fragaria × ananassa*	Strawberry	USA, FL	W. Turechek	-	-	-
Xanthomonas fragariae	Xf3	-	-	-	*Fragaria × ananassa*	Strawberry	USA, NC	W. Turechek	-	-	-
Xanthomonas fragariae	Xf128	-	-	-	*Fragaria × ananassa*	Strawberry	Canada	W. Turechek	-	-	-
Xanthomonas hortorum pv. *pelargonii*	LMG 7229	Xg4; ATCC 11057; ICPB XG4; NRRL B-1011	-	-	*Geranium pratense*	Meadow cranesbill	-	BCCM LMG	-	-	-
Xanthomonas vesicatoria	NRRL B-65404	TSC	-	-	*Solanum lycopersicum*	Tomato	USA	NRRL	-	-	-
Xanthomonas vesicatoria	LMG 911[T]	NCPPB 422; PDDCC 63; ICMP 63	-	-	*Solanum lycopersicum*	Tomato	New Zealand	BCCM LMG	-	-	-

[a]Assay 1 detects *R. pseudosolanacearum* (FAM) and was run in the QuantStudio™ 5 Real-Time PCR System; Assay 2 differentiates phylotypes I (FAM) and III (HEX) and was run in the CFX Opus Real-Time PCR System.

[b]B. Swingle – USDA Agricultural Research Service (ARS) Ithaca, NY; BCCM LMG - Belgian Coordinated Collections of Microorganisms Laboratory of Microbiology of Ghent University; C. Allen – University of Wisconsin-Madison Department of Plant Pathology; J. Leach – Colorado State University Department of Agricultural Biology; M. Arif - University of Hawai‘i Department of Plant & Environmental Protection Sciences; M.J.C. Pel - Netherlands Institute for Vectors, Invasive plants and Plant health (NIVIP); NRRL - USDA-ARS Northern Regional Research Laboratory; P. Elia - USDA-ARS Soybean Genomics & Improvement Laboratory; PPCDL - USDA APHIS PPQ S&T Plant Pathogen Confirmatory Diagnostics Laboratory; Q. Huang - USDA-ARS Floral and Nursery Plants Research Unit, U.S. National Arboretum; S. Miller – The Ohio State University Department of Plant Pathology; V. Stockwell - USDA-ARS Corvallis, OR; W. Turechek - United States Horticultural Research Lab (USHRL), Fort Pierce, FL.

Table S3. Small-scale specificity evaluation of portable assays.[a]

Strain	Phylotype	Seq	Host	Location	AmpliFire			Genie III			
						Assay 2			Assay 3		
					Assay 1	FAM (I)	HEX (III)	Assay 1	FAM (I)	ATTO (III)	Assay 4
202402-02	I	30	Ginger	NC, USA	+	+	-	+	+	-	+
UW361	I	12	Mulberry	China	+	+	-	+	+	-	+
UW386	III	23	Soil	Nigeria	+	-	+	+	-	+	+
UW472	III	43	Potato	Nigeria	+	-	+	+	-	+	+
CFBP3059	III	23	Eggplant	Burkina Faso	+	-	+	+	-	+	+
J25	III	20	Potato	Kenya	+	-	+	+	-	+	+
JT525	III	19	Geranium	Réunion	+	-	+	+	-	+	+
LMG 9673^T	III	19	Geranium	Réunion	+	-	+	+	-	+	+
202401-06	IIC	7	Tomato	GA, USA	-	-	-	-	-	-	-
CFBP8695	IIB	1	Potato	Iran	-	-	-	-	-	-	-
MolK2	IIB	3	Banana	Phillippines	-	-	-	-	-	-	-
LMG 27703^T	IV	10	Tomato	Indonesia	-	-	-	-	-	-	-
LMG 10661^T	IV	9	Clove	Indonesia	-	-	-	-	-	-	-

[a]Assay 1 detects *R. pseudosolanacearum* (FAM); Assay 2 detects and differentiate *R. pseudosolanacearum* phylotypes I (FAM) and III (HEX); Assay 3 detects and differentiate *R. pseudosolanacearum* phylotypes I (FAM) and III (ATTO590); Assay 4 is a colorimetric LAMP assay that detects *R. pseudosolanacearum*.

Table S4. Diagnostic samples used in this study and corresponding 16S rRNA real-time PCR and LAMP data.[a]

Organism	Sample ID	Phylotype	Sequevar	Host	Host (Common)	Location	16S C_t value	Assay 1	Assay 2	
									Phylotype I	Phylotype III
Ralstonia pseudosolanacearum	201702-02	I	18	*Solanum melongena*	Eggplant	Guam	19.00	+	+	-
Ralstonia pseudosolanacearum	201702-04	I	18	*Solanum melongena*	Eggplant	Guam	19.00	+	+	-
Ralstonia pseudosolanacearum	201702-07	I	18	*Capsicum annuum*	Pepper	Guam	36.00	+	+	-
Ralstonia pseudosolanacearum	202201-01	I	30	Zingiber officinale	Ginger	NY, USA	14.57	+	+	-
Ralstonia pseudosolanacearum	202301-01	I	13	*Kalanchoe sp.*	Widow's-thrill	NC, USA	13.08	+	+	-
Ralstonia pseudosolanacearum	202302-01	I	30	*Ostcospennum sp.*	African daisy	CA, USA	13.86	+	+	-
Ralstonia pseudosolanacearum	202302-02	I	30	*Ostcospennum sp.*	African daisy	CA, USA	15.98	+	+	-
Ralstonia pseudosolanacearum	202405-01	I	30	*Zingiber officinale*	Ginger	MN, USA	15.65	+	+	-
Ralstonia pseudosolanacearum	202405-02	I	30	*Zingiber officinale*	Ginger	MN, USA	17.25	+	+	-
Ralstonia pseudosolanacearum	202401-01	I	30	*Solanum lycopersicum*	Tomato	RI, USA	15.65	+	+	-
Ralstonia pseudosolanacearum	202402-01	I	30	*Zingiber officinale*	Ginger	NJ, USA	17.27	+	+	-
Ralstonia pseudosolanacearum	202401-02	I	30	*Zingiber officinale*	Ginger	MN, USA	15.75	+	+	-
Ralstonia pseudosolanacearum	202401-03	I	30	*Zingiber officinale*	Ginger	NC, USA	12.19	+	+	-
Ralstonia pseudosolanacearum	202402-02	I	30	*Lactuca sativa*	Lettuce	NC, USA	12.35	+	+	-
Ralstonia pseudosolanacearum	202401-04	I	30	*Zingiber officinale*	Ginger	IL, USA	24.37	+	+	-
Ralstonia solanacearum	202402-03	IIA	38	*Solanum lycopersicum*	Tomato	GA, USA	14.58	-	-	-
Ralstonia solanacearum	201601-01	IIB	4	*Portulaca sp.*	Purslanes	IL, USA	12.00	-	-	-
Ralstonia solanacearum	202001-01	IIB	1	*Pelargonium sp.*	Geranium	MI, USA	17.48	-	-	-
Ralstonia solanacearum	202001-02	IIB	1	*Pelargonium sp.*	Geranium	MI, USA	13.98	-	-	-
Ralstonia solanacearum	202002-03	IIB	1	*Pelargonium sp.*	Geranium	OR, USA	24.07	-	-	-
Ralstonia solanacearum	201301-01	IIC	7	*Solanum lycopersicum*	Tomato	GA, USA	11.00	-	-	-
Ralstonia solanacearum	201301-02	IIC	7	*Solanum lycopersicum*	Tomato	GA, USA	33.00	-	-	-
Ralstonia solanacearum	202302-03	IIC	7	*Solanum lycopersicum*	Tomato	SC, USA	20.32	-	-	-
Ralstonia solanacearum	202401-05	IIC	7	*Vaccinium sp.*	Blueberry	NC, USA	12.96	-	-	-
Ralstonia solanacearum	202401-06	IIC	7	*Solanum lycopersicum*	Tomato	GA, USA	12.05	-	-	-
Ralstonia solanacearum	202401-07	IIC	7	*Solanum lycopersicum*	Tomato	GA, USA	21.83	-	-	-
Ralstonia solanacearum	202502-01	IIC	7	*Solanum lycopersicum*	Tomato	GA, USA	11.63	-	-	-

[a]Assay 1 detects *R. pseudosolanacearum* using a FAM probe and was run in the QuantStudio™ 5 Real-Time PCR System; Assay 2 detects and differentiate *R. pseudosolanacearum* phylotypes I (FAM) and III (HEX) and was run in the CFX Opus Real-Time PCR System.

Table S5. Small-scale diagnostic sensitivity and specificity evaluation of portable assays.[a]

Strain	Phylotype	Seq	Host	Location	AmpliFire			Genie III			Color
					FAM	FAM (I)	HEX (III)	FAM	FAM (I)	ATTO (III)	
202302-01	I	13	African daisy	CA, USA	+	+	-	+	+	-	+
201702-04	I	18	Eggplant	Guam	+	+	-	+	+	-	+
201702-07	I	18	Pepper	Guam	+	NT	NT	+	NT	NT	NT
202402-01	I	30	Ginger	NJ, USA	+	+	-	+	+	-	+
202601-01	I	30	Tomato	HI, USA	+	+	-	+	+	-	+
202601-02	I	30	Eggplant	HI, USA	+	+	-	+	+	-	+
202601-03	I	34	Eggplant	Guam	+	+	-	+	+	-	+
202601-04	I	34	Eggplant	Guam	+	+	-	+	+	-	+
202601-05	I	34	Eggplant	Guam	+	+	-	+	+	-	+
202402-03	IIA	38	Tomato	GA, USA	-	-	-	-	-	-	-
202001-02	IIB	1	Geranium	MI, USA	-	-	-	-	-	-	-
202401-05	IIC	7	Blueberry	NC, USA	-	-	-	-	-	-	-
NTC	-	-	-	-	-	-	-	-	-	-	-

[a]NT = not tested; Assay 1 detects *R. pseudosolanacearum* (FAM); Assay 2 detects and differentiate *R. pseudosolanacearum* phylotypes I (FAM) and III (HEX); Assay 3 detects and differentiate *R. pseudosolanacearum* phylotypes I (FAM) and III (ATTO590); Assay 4 is a colorimetric LAMP assay that detects *R. pseudosolanacearum*.

Table S6. Intermediate precision results for three operators testing the same blind sample panel.

Well	Sample (Phylotype)	Conc.	N	ISO-004nd (AmpliFire)					ISO-004-VIS	
				FAM Duplex		FAM/HEX Duplex			Duplex	
				Expected Result	% Agree	Expected Result (FAM)	Expected Result (HEX)	% Agree	Expected Result	% Agree
1	202402-02 (I) cells	10 CFU/µl	9	+	100.00%	+	-	100.00%	+	100.00%
2	UW685 (III) cells	10 CFU/µl	9	+	100.00%	-	+	100.00%	+	100.00%
3	Molk2 (II)	10 pg/µl	9	-	100.00%	-	-	100.00%	-	100.00%
4	LMG 27703^T (IV)	10 pg/µl	9	-	100.00%	-	-	100.00%	-	100.00%
5	UW386 (III)	10 pg/µl	9	+	100.00%	-	+	100.00%	+	100.00%
6	CP synth ctrl	100 ag/µl	9	+	100.00%	+	-	100.00%	+	100.00%
7	HP synth ctrl	100 ag/µl	9	+	100.00%	-	+	100.00%	+	100.00%
8	NTC	-	9	-	100.00%	-	-	100.00%	-	100.00%

Well	Sample (Phylotype)	Conc.	N	ISO-004nd (Genie III)				
				FAM Duplex		FAM/ATTO Duplex		
				Expected Result	% Agree	Expected Result (FAM)	Expected Result (ATTO590)	% Agree
1	202402-02 (I) cells	10 CFU/µl	9	+	100.00%	+	-	100.00%
2	UW685 (III) cells	10 CFU/µl	9	+	100.00%	-	+	100.00%
3	Molk2 (II)	10 pg/µl	9	-	100.00%	-	-	100.00%
4	LMG 27703^T (IV)	10 pg/µl	9	-	100.00%	-	-	100.00%
5	UW386 (III)	10 pg/µl	9	+	100.00%	-	+	100.00%
6	CP synth ctrl	100 ag/µl	9	+	100.00%	+	-	100.00%
7	HP synth ctrl	100 ag/µl	9	+	100.00%	-	+	100.00%
8	NTC	-	9	-	100.00%	-	-	100.00%

Table S7. LAMP assay data for inoculated plants.

Host	Sample[a]	Strain	Phylotype	Lab[b]	DI[c]	16S	AmpliFire®			Genie® III			Assay 4[d]
							Assay 1	Assay 2		Assay 1	Assay 3		
							(FAM)	FAM (I)	HEX (III)	(FAM)	FAM (I)	ATTO590 (III)	
Geranium	Ge-H-1	-	-	ARS	0	-	-	-	-	-	-	-	-
Geranium	Ge-H-2	-	-	ARS	0	-	-	-	-	-	-	-	NT
Geranium	Ge-H-3	-	-	ARS	0	-	-	-	-	-	-	-	NT
Geranium	Ge-H-4	-	-	ARS	0	-	-	-	-	-	-	-	-
Geranium	Ge-I-1	202402-02	I	PPCDL	0	-	-	-	-	-	-	-	-
Geranium	Ge-I-2	202402-02	I	PPCDL	0	-	-	-	-	-	-	-	-
Geranium	Ge-III-1a	UW685	III	PPCDL	3	16.65	+	-	+	+	-	+	+
Geranium	Ge-III-1b	UW685	III	PPCDL	3	17.43	+	-	+	+	-	+	+
Geranium	Ge-III-2a	UW685	III	PPCDL	3	16.13	+	-	+	+	-	+	+
Geranium	Ge-III-2b	UW685	III	PPCDL	3	15.29	+	-	+	+	-	+	+
Geranium	Ge-H-1	-	-	PPCDL	0	-	-	-	-	-	-	-	-
Geranium	Ge-H-2	-	-	PPCDL	0	-	-	-	-	-	-	-	-
Ginger	Gi-I-1	202402-02	I	PPCDL	0	-	-	-	-	-	-	-	-
Ginger	Gi-I-2	202402-02	I	PPCDL	0	-	-	-	-	-	-	-	-
Ginger	Gi-III-1	UW685	III	PPCDL	0	-	-	-	-	-	-	-	-
Ginger	Gi-III-2	UW685	III	PPCDL	0	-	-	-	-	-	-	-	-
Ginger	Gi-H-1	-	-	PPCDL	0	-	-	-	-	-	-	-	-
Ginger	Gi-H-2	-	-	PPCDL	0	-	-	-	-	-	-	-	-
Pepper	Pe-I-1	202402-02	I	PPCDL	0	-	-	-	-	-	-	-	-
Pepper	Pe-I-2	202402-02	I	PPCDL	0	-	-	-	-	-	-	-	-
Pepper	Pe-III-1	UW685	III	PPCDL	0	-	-	-	-	-	-	-	-
Pepper	Pe-III-2	UW685	III	PPCDL	0	-	-	-	-	-	-	-	-
Pepper	Pe-H-1	-	-	PPCDL	0	-	-	-	-	-	-	-	-
Pepper	Pe-H-2	-	-	PPCDL	0	-	-	-	-	-	-	-	-
Potato	Po-I-1	202402-02	I	PPCDL	0	-	-	-	-	-	-	-	-
Potato	Po-I-2	202402-02	I	PPCDL	0	-	-	-	-	-	-	-	-
Potato	Po-III-1a	UW685	III	PPCDL	4	14.87	+	-	+	+	-	+	+
Potato	Po-III-1b	UW685	III	PPCDL	4	16.45	+	-	+	+	-	+	+
Potato	Po-III-2	UW685	III	PPCDL	0	36.47	+	-	+	+	-	+	+
Potato	Po-H-1	-	-	PPCDL	0	-	-	-	-	-	-	-	-
Potato	Po-H-2	-	-	PPCDL	0	-	-	-	-	-	-	-	-
Rose	R1-PD-1	PD7123	I	PPCDL	0	-	-	-	-	-	-	-	-
Rose	R1-PD-2	PD7123	I	PPCDL	0	-	-	-	-	-	-	-	NT
Rose	R1-PD-3	PD7123	I	PPCDL	0	-	-	-	-	-	-	-	NT
Rose	R1-PD-4	PD7123	I	PPCDL	0	NT	NT	NT	NT	NT	NT	NT	NT
Rose	R1-PD-5	PD7123	I	PPCDL	0	NT	NT	NT	NT	NT	NT	NT	NT
Rose	R1-PD-6	PD7123	I	PPCDL	0	-	-	-	-	-	-	-	-
Rose	R1-UW-1	UW764	I	PPCDL	0	NT	NT	NT	NT	NT	NT	NT	NT
Rose	R1-UW-2	UW764	I	PPCDL	0	NT	NT	NT	NT	NT	NT	NT	NT
Rose	R1-UW-3	UW764	I	PPCDL	0	-	-	-	-	-	-	-	-
Rose	R1-UW-4	UW764	I	PPCDL	0	-	-	-	-	-	-	-	NT
Rose	R1-UW-5	UW764	I	PPCDL	0	-	-	-	-	-	-	-	-
Rose	R1-UW-6	UW764	I	PPCDL	0	NT	NT	NT	NT	NT	NT	NT	NT

Rose	R1-H-1	-	-	PPCDL	0	-	-	-	-	-	-	-	-
Rose	R1-H-2	-	-	PPCDL	0	-	-	-	-	-	-	-	NT
Rose	R1-H-3	-	-	PPCDL	0	-	-	-	-	-	-	-	NT
Rose	R1-H-4	-	-	PPCDL	0	-	-	-	-	-	-	-	-
Rose[e]	R2-PD-1a	PD7123	I	PPCDL	0	25.86	+	+	-	+	+	-	+
Rose[e]	R2-PD-1b	PD7123	I	PPCDL	0	28.00	+	+	-	+	+	-	+
Rose[e]	R2-PD-1c	PD7123	I	PPCDL	0	24.83	+	+	-	+	+	-	+
Rose	R2-PD-2a	PD7123	I	PPCDL	0	-	-	-	-	-	-	-	NT
Rose	R2-PD-2b	PD7123	I	PPCDL	0	-	-	-	-	-	-	-	NT
Rose	R2-PD-3a	PD7123	I	PPCDL	0	-	-	-	-	-	-	-	NT
Rose	R2-PD-3b	PD7123	I	PPCDL	0	-	-	-	-	-	-	-	NT
Rose	R2-PD-4a	PD7123	I	PPCDL	0	-	-	-	-	-	-	-	NT
Rose	R2-PD-4b	PD7123	I	PPCDL	0	-	-	-	-	-	-	-	NT
Rose	R2-PD-5a	PD7123	I	PPCDL	0	-	-	-	-	-	-	-	NT
Rose	R2-PD-5b	PD7123	I	PPCDL	0	-	-	-	-	-	-	-	NT
Rose	R2-PD-6a	PD7123	I	PPCDL	0	-	-	-	-	-	-	-	NT
Rose	R2-PD-6b	PD7123	I	PPCDL	0	-	-	-	-	-	-	-	NT
Rose	R2-PD-7a	PD7123	I	PPCDL	0	-	-	-	-	-	-	-	NT
Rose[e]	R2-PD-7b	PD7123	I	PPCDL	0	32.34	+	+	-	+	+	-	+
Rose[e]	R2-PD-7c	PD7123	I	PPCDL	0	28.99	+	+	-	+	+	-	+
Rose	R2-PD-8a	PD7123	I	PPCDL	0	-	-	-	-	-	-	-	NT
Rose	R2-PD-8b	PD7123	I	PPCDL	0	-	-	-	-	-	-	-	NT
Rose	R2-H-1	PD7123	I	PPCDL	0	-	-	-	-	-	-	-	-
Rose	R2-H-2	PD7123	I	PPCDL	0	-	-	-	-	-	-	-	-
Rose	R3-PD-1	PD7123	I	PPCDL	0	NT	NT	NT	NT	NT	NT	NT	NT
Rose[e]	R3-PD-2	PD7123	I	PPCDL	0	33.11	-	-	-	-	-	-	-
Rose[e]	R3-PD-3	PD7123	I	PPCDL	0	35.91	-	-	-	-	-	-	-
Rose	R3-PD-4	PD7123	I	PPCDL	0	-	-	-	-	-	-	-	NT
Rose	R3-PD-5	PD7123	I	PPCDL	0	-	-	-	-	-	-	-	NT
Rose	R3-PD-6	PD7123	I	PPCDL	0	NT	NT	NT	NT	NT	NT	NT	NT
Rose	R3-PD-7	PD7123	I	PPCDL	0	-	-	-	-	-	-	-	-
Rose	R3-PD-8	PD7123	I	PPCDL	0	NT	NT	NT	NT	NT	NT	NT	NT
Rose	R3-UW-1	UW764	I	PPCDL	0	-	-	-	-	-	-	-	NT
Rose	R3-UW-2	UW764	I	PPCDL	0	NT	NT	NT	NT	NT	NT	NT	NT
Rose	R3-UW-3	UW764	I	PPCDL	0	NT	NT	NT	NT	NT	NT	NT	NT
Rose[e]	R3-UW-4	UW764	I	PPCDL	0	34.29	+	+	-	+	+	-	+
Rose	R3-UW-5	UW764	I	PPCDL	0	NT	NT	NT	NT	NT	NT	NT	NT
Rose	R3-UW-6	UW764	I	PPCDL	0	NT	NT	NT	NT	NT	NT	NT	NT
Rose	R3-UW-7	UW764	I	PPCDL	0	NT	NT	NT	NT	NT	NT	NT	NT
Rose	R3-UW-8	UW764	I	PPCDL	0	NT	NT	NT	NT	NT	NT	NT	NT
Rose	R3-H-1	-	-	PPCDL	0	-	-	-	-	-	-	-	-
Rose	R3-H-2	-	-	PPCDL	0	-	-	-	-	-	-	-	NT
Rose	R3-H-3	-	-	PPCDL	0	-	-	-	-	-	-	-	NT
Rose	R3-H-4	-	-	PPCDL	0	-	-	-	-	-	-	-	-
Tomato	T-121-1	Rs121	I	ARS	0	19.60	+	+	-	+	+	-	+
Tomato	T-121-2	Rs121	I	ARS	2	18.09	+	+	-	+	+	-	NT
Tomato	T-121-3	Rs121	I	ARS	2	18.83	+	+	-	+	+	-	NT

Tomato	T-121-4	Rs121	I	ARS	3	18.20	+	+	-	+	+	-	NT
Tomato	T-121-5	Rs121	I	ARS	3	19.00	+	+	-	+	+	-	NT
Tomato	T-121-6	Rs121	I	ARS	3	18.66	+	+	-	+	+	-	NT
Tomato	T-121-7	Rs121	I	ARS	4	17.23	+	+	-	+	+	-	NT
Tomato	T-121-8	Rs121	I	ARS	4	17.06	+	+	-	+	+	-	+
Tomato	T-262-1	Pss262	I	ARS	0	21.00	+	+	-	+	+	-	+
Tomato	T-262-2	Pss262	I	ARS	0	20.41	+	+	-	+	+	-	NT
Tomato	T-262-3	Pss262	I	ARS	1	19.99	+	+	-	+	+	-	NT
Tomato	T-262-4	Pss262	I	ARS	1	20.99	+	+	-	+	+	-	NT
Tomato	T-262-5	Pss262	I	ARS	1	19.84	+	+	-	+	+	-	NT
Tomato	T-262-6	Pss262	I	ARS	2	20.37	+	+	-	+	+	-	NT
Tomato	T-262-7	Pss262	I	ARS	3	19.21	+	+	-	+	+	-	NT
Tomato	T-262-8	Pss262	I	ARS	3	19.68	+	+	-	+	+	-	+
Tomato	T-1000-1	GMI1000	I	ARS	0	26.00	+	+	-	+	+	-	+
Tomato	T-1000-2	GMI1000	I	ARS	0	19.56	+	+	-	+	+	-	NT
Tomato	T-1000-3	GMI1000	I	ARS	2	19.15	+	+	-	+	+	-	NT
Tomato	T-1000-4	GMI1000	I	ARS	2	19.50	+	+	-	+	+	-	NT
Tomato	T-1000-5	GMI1000	I	ARS	4	19.68	+	+	-	+	+	-	NT
Tomato	T-1000-6	GMI1000	I	ARS	4	19.11	+	+	-	+	+	-	NT
Tomato	T-1000-7	GMI1000	I	ARS	4	19.49	+	+	-	+	+	-	NT
Tomato	T-1000-8	GMI1000	I	ARS	4	19.18	+	+	-	+	+	-	+
Tomato[f]	T-302-1	RUN302	II	ARS	4	20.24	+	+	-	+	+	-	+
Tomato[f]	T-302-2	RUN302	II	ARS	4	18.75	+	+	-	+	+	-	+
Tomato[f]	T-302-3	RUN302	II	ARS	4	18.01	+	+	-	+	+	-	+
Tomato[f]	T-302-4	RUN302	II	ARS	4	17.11	+	+	-	+	+	-	+
Tomato[f]	T-302-5	RUN302	II	ARS	4	18.06	+	+	-	+	+	-	+
Tomato[f]	T-302-6	RUN302	II	ARS	4	18.47	+	+	-	+	+	-	+
Tomato[f]	T-302-7	RUN302	II	ARS	4	19.20	+	+	-	+	+	-	+
Tomato[f]	T-302-8	RUN302	II	ARS	4	18.64	+	+	-	+	+	-	+
Tomato	T-H-1	-	-	ARS	0	-	-	-	-	-	-	-	-
Tomato	T-H-2	-	-	ARS	0	-	-	-	-	-	-	-	NT
Tomato	T-H-3	-	-	ARS	0	-	-	-	-	-	-	-	NT
Tomato	T-H-4	-	-	ARS	0	-	-	-	-	-	-	-	-
Tomato	T-I-1	202402-02	I	PPCDL	0	-	-	-	-	-	-	-	-
Tomato	T-I-2	202402-02	I	PPCDL	0	-	-	-	-	-	-	-	-
Tomato	T-III-1	UW685	III	PPCDL	0	17.37	+	-	+	+	-	+	+
Tomato	T-III-2	UW685	III	PPCDL	0	19.98	+	-	+	+	-	+	+
Tomato	T-H-1	-	-	PPCDL	0	-	-	-	-	-	-	-	-
Tomato	T-H-2	-	-	PPCDL	0	-	-	-	-	-	-	-	-

[a]Sample number reflects the host, infecting strain or pathotype, and the plant number. H designates “healthy” mock-inoculated plants. The use of a and b designates different shoot samples taken from the same plant. R1 = ‘Rio Samba’; R2 = Papa Meilland®; R3 = Pink Double Knock Out®.

[b]Designates the laboratory at which inoculations were performed. ARS – Agricultural Research Service; PPCDL – Plant Pathogen Confirmatory Diagnostics Laboratory.

[c]Disease Index - 0: Asymptomatic (No wilting); 1: 1-25% of leaves wilted; 2: 25-50% of leaves wilted; 3: 51-75% of leaves wilted; 4: 76-100% of leaves wilted.

[d]NT = Not Tested

[e]16S-positive rose samples did not produce positive LAMP results due to inhibition. Rose samples were cleaned using an alternate protocol; presented C_t values are post-cleaning.

[f]Tomato samples infected with RUN302 produced positive results by LAMP. The RUN302 culture used for inoculations was found to also contain an unknown Phylotype I contaminant strain. Results from four independent phylotype I-specific assays demonstrated the presence of phylotype I in the RUN302 culture, as well as the infected tomato.

Table S8. LAMP and corresponding 16S PCR assay data for inoculated rose plants after streaming, cleaning the stream with a commercial clean-up kit, and cleaning the stream with a field clean-up protocol.

Sample	Strain	Stream			Stream Cleaned (KIT)					Stream Cleaned (Field - Filter)				
		ToughMix			ToughMix		LUNA			ToughMix		LUNA		
		Mean	StDev	LAMP	Mean	StDev	Mean	StDev	LAMP	Mean	StDev	Mean	StDev	LAMP
1-1	PD7123	35.45	0.04	-	-	-	-	-	-	-	-	-	-	-
1-2	PD7123	30.96	0.96	-	-	-	36.58	0.11	-	-	-	-	-	-
1-3	PD7123	34.20	0.84	-	-	-	-	-	-	-	-	-	-	-
1-4	PD7123	-	-	-	-	-	-	-	-	NT	NT	NT	NT	NT
1-5	PD7123	-	-	-	-	-	-	-	-	NT	NT	NT	NT	NT
1-6	PD7123	-	-	-	-	-	36.53	-	-	-	-	-	-	-
2-1	UW764	-	-	-	-	-	-	-	-	NT	NT	NT	NT	NT
2-2	UW764	-	-	-	-	-	-	-	-	NT	NT	NT	NT	NT
2-3	UW764	-	-	-	-	-	39.26	0.87	-	-	-	-	-	-
2-4	UW764	-	-	-	-	-	36.94	0.29	+	-	-	-	-	-
2-5	UW764	38.86	-	-	-	-	-	-	-	-	-	-	-	-
2-6	UW764	-	-	-	-	-	-	-	-	NT	NT	NT	NT	NT
DK-1-1	PD7123	-	-	-	-	-	-	-	-	NT	NT	NT	NT	NT
DK-1-2	PD7123	33.61	0.18	-	-	-	37.82	0.59	-	33.11	1.37	-	-	-
DK-1-3	PD7123	34.79	0.24	-	-	-	34.40	0.25	-	35.91	-	-	-	-
DK-1-4	PD7123	36.04	0.34	-	-	-	-	-	-	-	-	-	-	-
DK-1-5	PD7123	29.71	0.48	-	-	-	34.45	1.11	+	-	-	-	-	-
DK-1-6	PD7123	-	-	-	-	-	-	-	-	NT	NT	NT	NT	NT
DK-1-7	PD7123	30.47	0.32	-	-	-	35.31	0.69	+	-	-	-	-	-
DK-1-8	PD7123	-	-	-	-	-	-	-	-	NT	NT	NT	NT	NT
DK2-1	UW764	-	-	-	-	-	37.71	0.85	-	-	-	-	-	-
DK2-2	UW764	-	-	-	-	-	-	-	-	NT	NT	NT	NT	NT
DK2-3	UW764	-	-	-	-	-	-	-	-	NT	NT	NT	NT	NT
DK2-4	UW764	35.47	-	-	-	-	35.25	0.47	+	33.18	1.47	35.40	0.38	+
DK2-5	UW764	-	-	-	-	-	-	-	-	NT	NT	NT	NT	NT
DK2-6	UW764	-	-	-	-	-	-	-	-	NT	NT	NT	NT	NT
DK2-7	UW764	-	-	-	-	-	-	-	-	NT	NT	NT	NT	NT
DK2-8	UW764	-	-	-	-	-	-	-	-	NT	NT	NT	NT	NT
H-1	-	-	-	-	NT	NT	NT	NT	-	NT	NT	NT	NT	NT
H-2	-	-	-	-	NT	NT	NT	NT	-	NT	NT	NT	NT	NT
H-3	-	-	-	-	NT	NT	NT	NT	-	NT	NT	NT	NT	NT
H-4	-	-	-	-	NT	NT	NT	NT	-	NT	NT	NT	NT	NT
DK-H-1	-	-	-	-	NT	NT	NT	NT	-	NT	NT	NT	NT	NT
DK-H-2	-	-	-	-	NT	NT	NT	NT	-	NT	NT	NT	NT	NT
DK-H-3	-	-	-	-	NT	NT	NT	NT	-	NT	NT	NT	NT	NT
DK-H-4	-	-	-	-	NT	NT	NT	NT	-	NT	NT	NT	NT	NT

NT = Not tested

Table S9. Bacterial strains and associated genome accessions used for comparative analysis to find unique diagnostic targets.

Organism	Phylotype	Strain ID	Accession
Ralstonia pseudosolanacearum	I	GMI1000	GCA_000009125.1
Ralstonia pseudosolanacearum	III	RUN2340	GCF_029220045.1
Ralstonia solanacearum	IIA	CFBP2957	GCA_029220025.1
Ralstonia solanacearum	IIA	IBSBF 2570	GCA_003590585.2
Ralstonia solanacearum	IIA	K60	GCA_002251695.1
Ralstonia solanacearum	IIA	SFC	GCA_003590625.2
Ralstonia solanacearum	IIB	CCRMRsB7	GCA_014210345.1
Ralstonia solanacearum	IIB	IPO1609	GCA_001050995.1
Ralstonia solanacearum	IIB	Po82	GCA_000215325.1
Ralstonia solanacearum	IIB	UY031	GCA_001299555.1
Ralstonia syzygii	IV	PSI07	GCA_037101365.1
Ralstonia syzygii	IV	R24	GCA_029219965.1
Ralstonia eutrophora	-	H16	GCA_000009285.2
Ralstonia eutrophora	-	JMP134	GCA_000203875.1
Ralstonia pickettii	-	12J	GCA_000020205.1
Ralstonia sp.	-	SET104	GCA_003851545.1
Ralstonia sp.	-	TCR112	GCA_007997035.1
[Pseudomonas] *boreopolis*	-	JCM 13306	GCA_014654535.1
[Pseudomonas] *geniculata*	-	ATCC 19374	GCA_001431625.1
[Pseudomonas] *hibiscicola*	-	ATCC 19867	GCA_000382065.1
Acidovorax avenae subsp. *avenae*	-	ATCC 19860	GCA_000176855.2
Acidovorax citrulli	-	AAC00-1	GCA_000015325.1
Acidovorax ebreus	-	TPSY	GCA_000022305.1
Acidovorax sp.	-	JS42	GCA_000015545.1
Acidovorax sp.	-	KKS102	GCA_000302535.1
Burkholderia ambifaria	-	IOP40-10	GCA_000181975.1
Burkholderia anthina	-	DSM 16086	GCA_016836725.1
Burkholderia anthina	-	LMG 20980	GCA_902830395.1
Burkholderia bryophila	-	376MFSha3.1	GCA_000383275.1
Burkholderia cenocepacia	-	DWS 37E-2	GCA_000764955.1
Burkholderia cenocepacia	-	K56-2	GCA_014357995.1
Burkholderia cepacia	-	ATCC 25416	GCA_003546465.1
Burkholderia dabaoshanensis	-	GIMN1.004	GCA_002879885.1
Burkholderia diffusa	-	DSM 23434	GCA_016836765.1
Burkholderia dolosa	-	AU0158	GCA_000152585.1
Burkholderia gladioli	-	ATCC 10248	GCA_000959725.1
Burkholderia glumae	-	LMG 2196	GCA_000300755.1
Burkholderia humptydooensis	-	MSMB43	GCA_001513745.1
Burkholderia lata	-	LK13	GCA_001038835.1
Burkholderia mallei	-	NCTC 10229	GCA_000015605.1
Burkholderia multivorans	-	ATCC 17616	GCA_000010545.1
Burkholderia plantarii	-	ATCC 43733	GCA_001411805.1
Burkholderia plantarii	-	LMG 9035	GCA_902832905.1
Burkholderia pseudomallei	-	NCTC 13178	GCA_000511895.1
Burkholderia pyrrocinia	-	LMG 14191	GCA_902832895.1
Burkholderia reimsis	-	BE51	GCA_003294055.1
Burkholderia rhizoxinica	-	HKI 454	GCA_000198775.1
Burkholderia sp.	-	JPY347	GCA_000373005.1
Burkholderia sp.	-	TJI49	GCA_000191945.2

Burkholderia stabilis	-	ATCC BAA-67	GCA_001742165.1
Burkholderia terrae	-	BS001	GCA_000265115.1
Burkholderia thailandensi	-	MSMB121	GCA_000385525.1
Burkholderia vietnamiensis	-	LMG 10929	GCA_902830295.1
Burkholderia xenovorans	-	LB400	GCA_000013645.1
Candidatus Burkholderia kirkii	-	UZHbot1	GCA_000234195.1
Clavibacter michiganensis	-	LMG7333	GCA_021216655.1
Clavibacter michiganensis subsp. *insidiosus*	-	ATCC 10253	GCA_003076355.1
Clavibacter michiganensis subsp. *insidiosus*	-	R1-1	GCA_000958465.1
Clavibacter michiganensis subsp. *michiganensis*	-	A4758	GCA_023520835.1
Clavibacter michiganensis subsp. *michiganensis*	-	A5747*	GCA_023520815.1
Clavibacter michiganensis subsp. *michiganensis*	-	Cmm_21	GCA_028623025.1
Clavibacter michiganensis subsp. *michiganensis*	-	CMM04	GCA_028622935.1
Clavibacter michiganensis subsp. *michiganensis*	-	CMM09	GCA_020616195.1
Clavibacter michiganensis subsp. *michiganensis*	-	CMM39	GCA_020616235.1
Clavibacter michiganensis subsp. *michiganensis*	-	CMM84	GCA_020616175.1
Clavibacter michiganensis subsp. *michiganensis*	-	MSF322	GCA_011995665.1
Clavibacter michiganensis subsp. *michiganensis*	-	N.P.	GCA_022983255.1
Clavibacter michiganensis subsp. *michiganensis*	-	NCPPB 382	GCA_000063485.1
Clavibacter michiganensis subsp. *michiganensis*	-	UF1	GCA_009739655.1
Clavibacter michiganensis subsp. *michiganensis*	-	VL527	GCA_011995885.1
Clavibacter michiganensis subsp. *michiganensis*	-	VQ143	GCA_019263785.1
Clavibacter michiganensis subsp. *michiganensis*	-	VQ28	GCA_019263765.1
Clavibacter michiganensis subsp. *tessellarius*	-	ATCC 33566	GCA_021922985.1
Cupriavidus basilensis	-	OR16	GCA_000243095.2
Cupriavidus campinensis	-	LMG 19282	GCA_905397395.1
Cupriavidus campinensis	-	MJ1	GCF_008271425.1
Cupriavidus campinensis	-	S14E4C	GCA_007572485.1
Cupriavidus metallidurans	-	BS1	GCA_003260185.2
Cupriavidus metallidurans	-	CH34	GCA_000196015.1
Cupriavidus metallidurans	-	NA4	GCA_000709045.1
Cupriavidus necator	-	N-1	GCA_000219215.1
Cupriavidus sp.	-	amp6	GCA_000426345.1
Cupriavidus sp.	-	BIS7	GCA_000292345.1
Cupriavidus sp.	-	HMR-1	GCA_000319775.1
Cupriavidus sp.	-	HPC(L)	GCA_000307735.3
Cupriavidus sp.	-	UYMMa02A	GCA_001725945.1
Cupriavidus sp.	-	UYPR2.512	GCA_000379565.1
Cupriavidus sp.	-	WS	GCA_000395345.1
Cupriavidus taiwanensis	-	LMG19424	GCA_000069785.1
Delftia acidovorans	-	SPH-1	GCA_000018665.1
Halopseudomonas gallaeciensis	-	V113	GCA_003444685.1
Halopseudomonas pachastrellae	-	CCUG 46540	GCA_001989375.1
Halopseudomonas sp.	-	RR6	GCA_021545785.1
Pantoea agglomerans	-	299R	GCA_000330765.1
Pantoea agglomerans	-	FDAARGOS 1447	GCA_019048385.1
Pantoea agglomerans	-	IG1	GCA_000241285.2
Pantoea agglomerans	-	P10c	GCA_001288285.1
Pantoea agglomerans	-	P5	GCA_002157425.2
Pantoea agglomerans	-	Tx10	GCA_000475055.1
Pantoea alhagi	-	LTYR-11Z	GCA_002101395.1
Pantoea ananatis	-	LMG 20103	GCA_000025405.2

Pantoea ananatis	-	LMG 5342	GCA_000283875.1
Pantoea ananatis	-	PA13	GCA_000233595.1
Pantoea cypripedii	-	LMG 2657	GCA_002095535.1
Pantoea deleyi	-	LMG24200	GCF_022647325.1
Pantoea dispersa	-	EGD-AAK13	GCA_000465555.2
Pantoea sp.	-	A4	GCA_000295955.2
Pantoea sp.	-	Ap-959	GCA_011752575.1
Pantoea sp.	-	AS-PWVM4	GCA_000468095.1
Pantoea sp.	-	At-9b	GCA_000175935.2
Pantoea sp.	-	GM01	GCA_000282675.1
Pantoea sp.	-	Sc1	GCA_000255315.2
Pantoea sp.	-	Sl1_M5	GCA_000220605.2
Pantoea stewartii subsp. *stewartii*	-	DC283	GCA_000248395.2
Pantoea vagans	-	C9-1	GCA_000148935.1
Paraburkholderia agricolaris	-	BaQS159	GCA_009455635.1
Paraburkholderia aspalathi	-	LMG 27731	GCA_900116445.1
Paraburkholderia atlantica	-	JPY171	GCA_014200895.1
Paraburkholderia bonniea	-	BbQS859	GCA_009455625.1
Paraburkholderia caledonica	-	NBRC 102488	GCA_000685095.1
Paraburkholderia caribensis	-	DSM 13236^T	GCA_002902945.1
Paraburkholderia dilworthii	-	WSM3556	GCA_000472525.1
Paraburkholderia franconis	-	CNPSo 3157	GCA_009362735.1
Paraburkholderia fungorum	-	LMG 28183	GCA_003002115.1
Paraburkholderia graminis	-	LMG 18924	GCA_902859785.1
Paraburkholderia guartelaensis	-	CNPSo 3008	GCA_004353905.1
Paraburkholderia hayleyella	-	BhQS11	GCA_009455685.1
Paraburkholderia hospita	-	DSM 17164^T	GCA_002902965.1
Paraburkholderia kururiensis	-	JCM 10599	GCA_000616025.1
Paraburkholderia kururiensis subsp. *thiooxydans*	-	NBRC 107107	GCA_000739795.1
Paraburkholderia madseniana	-	RP11	GCA_009690905.1
Paraburkholderia megapolitana	-	LMG 23650	GCA_007556815.1
Paraburkholderia mimosarum	-	LMG 23256	GCA_000472825.1
Paraburkholderia oxyphila	-	NBRC 105797	GCA_000685075.1
Paraburkholderia phenazinium	-	LMG 2247	GCA_900100735.1
Paraburkholderia phenoliruptrix	-	BR3459a	GCA_000300095.1
Paraburkholderia phymatum	-	STM815	GCA_000020045.1
Paraburkholderia phytofirmans	-	PsJN	GCA_000020125.1
Paraburkholderia sabiae	-	LMG 24235	GCA_904848645.1
Paraburkholderia sacchari	-	LMG 19450	GCA_902833715.1
Paraburkholderia sp.	-	RAU2J	GCA_003634845.1
Paraburkholderia sprentiae	-	WSM5005	GCA_000473465.1
Paraburkholderia terrae	-	DSM 17804^T	GCA_002902925.1
Paraburkholderia tropica	-	LMG 22274	GCA_900109265.1
Paraburkholderia tuberum	-	WSM4176	GCA_000372945.1
Paraburkholderia youngii	-	JPY162	GCA_014200925.1
Pseudomonas aeruginosa	-	ATCC 14886	GCA_000297275.1
Pseudomonas alcaliphila	-	JAB1	GCA_001941865.1
Pseudomonas alkylphenolica	-	KL28	GCA_000746525.1
Pseudomonas allokribbensis	-	IzPS23	GCF_014863605.1
Pseudomonas amygdali pv. *hibisci*	-	ICMP9623	GCA_001400395.1
Pseudomonas asiatica	-	P1	GCA_028752155.1
Pseudomonas atacamensis	-	M7D1	GCA_004801935.1

Pseudomonas azotoformans	-	F77	GCA_002007785.1
Pseudomonas baetic	-	LMG 25716	GCA_002813455.1
Pseudomonas balearica	-	DSM 6083	GCA_000818015.1
Pseudomonas brassicacearum	-	JCM 11938	GCA_012034345.1
Pseudomonas brassicacearum subsp. *brassicacearum*	-	CCUG 51508	GCA_008801605.1
Pseudomonas brassicacearum subsp. *neoaurantiaca*	-	CDVBN10	GCA_013608025.1
Pseudomonas brenneri	-	JCM 13307	GCA_014646715.1
Pseudomonas campi	-	S1-A32-2	GCA_013200955.2
Pseudomonas carnis	-	-	GCA_902329575.1
Pseudomonas chloritidismutans	-	AW-1	GCA_000495915.1
Pseudomonas chlororaphis	-	ATCC 17415	GCA_003851145.1
Pseudomonas chlororaphis	-	DSM 50083	GCA_016803445.1
Pseudomonas chlororaphis subsp. *aureofaciens*	-	C50	GCA_003851385.1
Pseudomonas chlororaphis subsp. *chlororaphis*	-	GP72	GCA_000237045.2
*Pseudomonas chlororaphi*s subsp. *piscium*	-	ATCC 17411	GCA_003850385.1
Pseudomonas citronellolis	-	P3B5	GCA_001586155.1
Pseudomonas corrugata	-	DSM 7228	GCA_001269905.1
Pseudomonas denitrificans	-	ATCC 13867	GCA_000349845.1
Pseudomonas entomophila	-	L48	GCA_000026105.1
Pseudomonas extremaustralis	-	14-3b	GCA_000242115.2
Pseudomonas fluorescens	-	BRIP34879	GCA_000334015.1
Pseudomonas fluorescens	-	NCIMB 11764	GCA_000293885.3
Pseudomonas fluorescens	-	PA4C2	GCA_000785395.1
Pseudomonas fluorescens	-	SS101	GCA_000263675.2
Pseudomonas fragi	-	B25	GCA_000250615.1
Pseudomonas furukawaii	-	KF707	GCA_002355475.1
Pseudomonas gessardii	-	DSM 17152	GCA_001983165.1
Pseudomonas glycinae	-	MS586	GCA_001594225.2
Pseudomonas gozinkensis	-	IzPS32d	GCF_014863585.1
Pseudomonas graminis	-	PDD-13b-3	GCA_002093745.2
Pseudomonas granadensis	-	LMG 27940	GCA_900105485.1
Pseudomonas grimontii	-	DSM 17515	GCA_007858185.1
Pseudomonas guangdongensis	-	CCTCC 2012022	GCA_900105885.1
Pseudomonas helmanticensis	-	BIGb0525	GCA_004365765.1
Pseudomonas hydrolytica	-	DSWY01	GCA_021495345.2
Pseudomonas indica	-	PIC105	GCA_002286725.1
Pseudomonas iranensis	-	SWRI54	GCA_014268585.2
Pseudomonas izuensis	-	LAB-08	GCA_002356535.2
Pseudomonas karstica	-	CCM 7891	GCA_009707515.1
Pseudomonas kermanshahensis	-	Mr36	GCA_029215585.1
Pseudomonas khorasanensis	-	SWRI153	GCA_014268505.2
Pseudomonas kilonensis	-	DSM 13647	GCA_001269885.1
Pseudomonas knackmussii	-	B13	GCF_000689415.1
Pseudomonas koreensis	-	DSM 16610	GCA_012986595.1
Pseudomonas kurunegalensis	-	ANKC.G2	GCA_030291635.1
Pseudomonas lalkuanensis	-	PE08	GCA_008807375.1
Pseudomonas lini	-	DSM 16768	GCA_001042905.1
Pseudomonas linyingensis	-	LMG 25967	GCA_900109175.1
Pseudomonas mandelii	-	DSM 17967	GCA_007858265.1
Pseudomonas marginalis	-	ICMP 3553	GCA_001645105.1
Pseudomonas marincola	-	YSy11	GCA_900682675.2
Pseudomonas mendocina	-	GU	GCA_015645415.1

Pseudomonas migulae	-	NBRC 103157	GCA_002091715.1
Pseudomonas monteilii	-	SB3078	GCA_000510285.1
Pseudomonas moraviensis	-	BS3668	GCA_900105805.1
Pseudomonas mosselii	-	DSM 17497	GCA_000621225.1
Pseudomonas mucidolens	-	LMG 2223	GCA_900106045.1
Pseudomonas multiresinivorans populi	-	-	GCA_012971725.1
Pseudomonas ogarae	-	1855-344	GCA_000968575.1
Pseudomonas oleovorans	-	T9AD	GCA_900626055.2
Pseudomonas oryzae	-	KCTC 32247	GCA_900104805.1
Pseudomonas otitidis	-	MrB4	GCA_011397855.1
Pseudomonas parafulva	-	JBCS1880	GCA_003410295.1
Pseudomonas phenolilytica	-	RBPA9	GCA_021432765.1
Pseudomonas plecoglossicida	-	NyZ12	GCA_000831585.1
Pseudomonas protegens	-	PPRAR02	GCA_017923275.1
Pseudomonas psychrophila	-	HA-4	GCA_000282975.1
Pseudomonas psychrotolerans	-	L19	GCA_000236825.2
Pseudomonas putida	-	ATCC 12633	GCA_024508115.1
Pseudomonas resinovorans	-	NBRC 106553	GCA_000412695.1
Pseudomonas rhodesiae	-	DSM 14020	GCA_007858255.1
Pseudomonas sagittaria	-	JCM 18195	GCA_900115715.1
Pseudomonas savastanoi pv. *phaseolicola*	-	ICMP2740	GCA_001400605.1
Pseudomonas savastanoi pv. *savastanoi*	-	NCPPB 3335	GCA_000164015.3
Pseudomonas siliginis	-	SWRI31	GCA_019145195.1
Pseudomonas simiae	-	WCS417	GCA_000698265.1
Pseudomonas sp.	-	B10	GCA_900156235.1
Pseudomonas sp.	-	LFM046	GCA_000949385.2
Pseudomonas sp.	-	UK4	GCA_000174915.1
Pseudomonas spelaei	-	CCM 7893	GCA_009724245.1
Pseudomonas stutzeri	-	DSM 10701	GCA_000279165.1
Pseudomonas synxantha	-	2-79	GCA_003851495.1
Pseudomonas syringae	-	PDD-32b-74	GCA_002157375.1
Pseudomonas syringae pv. *phaseolicola*	-	1448A	GCA_000012205.1
Pseudomonas syringae pv. *syringae*	-	MB03	GCA_001623415.1
Pseudomonas syringae pv. *tomato*	-	DC3000	GCA_000007805.1
Pseudomonas thivervalensis	-	DSM 13194	GCA_001269655.1
Pseudomonas triclosanedens	-	ZM23	GCA_026686735.1
Pseudomonas triticicola	-	SWRI88	GCA_019145375.1
Pseudomonas umsongensis	-	CY-1	GCA_012647205.1
Pseudomonas veronii	-	DSM 11331	GCA_001439695.1
Pseudomonas viridiflava	-	TA043	GCA_000452485.1
Pseudomonas yamanorum	-	LMG 27247	GCA_900105735.1
Pseudomonas zhaodongensis	-	NEAU-ST5-21	GCA_003696365.1
Pseudoxanthomonas dokdonensis	-	DSM 21858	GCA_001431405.1
Rathayibacter toxicus	-	SA03-04	GCA_018801185.1
Rhodopseudomonas palustris	-	TIE-1	GCA_000020445.1
Xanthomonas arboricola	-	CFBP 7622	GCA_002940405.1
Xanthomonas arboricola pv. *juglandis*	-	CFBP 2528	GCA_001013475.1
Xanthomonas axonopodis pv. *axonopodis*	-	LMG 982	GCA_001401595.1
Xanthomonas axonopodis pv. *begoniae*	-	CFBP2524	GCA_002940645.1
Xanthomonas axonopodis pv. *citrumelo*	-	F1	GCA_000225915.1
Xanthomonas campestris pv. *musacearum*	-	NCPPB 2251	GCA_001189905.2
Xanthomonas campestris pv. *vesicatoria*	-	85-10	GCA_000009165.1

Xanthomonas citri	-	LMG 7399	GCA_001401545.2
Xanthomonas citri pv. *anacardii*	-	CFBP 2913	GCF_002688625.1
Xanthomonas citri pv. *bilvae*	-	NCPPB 3213	GCA_001497855.1
Xanthomonas citri pv. *citri*	-	C40	GCA_040815775.1
Xanthomonas citri pv. *fuscans*	-	CFBP1815	GCA_900234415.1
Xanthomonas citri pv. *fuscans*	-	CFBP6990	GCA_900234485.1
Xanthomonas citri pv. *glycines*	-	ICMP5732	GCA_021474205.1
Xanthomonas citri pv. *malvacearum*	-	LMG761	GCA_002019255.1
Xanthomonas citri pv. *mangiferaeindicae*	-	LMG 941	GCA_000263335.1
Xanthomonas citri pv. *punicae*	-	LMG 859	GCA_006228545.1
Xanthomonas citri pv. *sesbaniae*	-	LMG867	GCA_020011255.1
Xanthomonas citri pv. *vignicola*	-	CFBP7112	GCA_002218265.1
Xanthomonas euroxanthea	-	1	GCA_903989455.1
Xanthomonas euvesicatoria	-	LMG 12749	GCA_001401675.2
Xanthomonas euvesicatoria pv. *allii*	-	CFBP 6369	GCF_000730305.1
Xanthomonas fuscans subsp. *aurantifolii*	-	ICPB 10535	GCA_000175155.1
Xanthomonas gardneri	-	ATCC 19865	GCA_000192065.2
Xanthomonas hortorum pv. *carotae*	-	M081	GCA_000505565.1
Xanthomonas hortorum pv. *gardneri*	-	CFBP 8163	GCA_012922265.1
Xanthomonas hortorum pv. *pelargoni*	-	CFBP 2533	GCA_903978215.1
Xanthomonas hortorum pv. *taraxaci*	-	CFBP 410	GCA_012922225.1
Xanthomonas hortorum pv. *vitians*	-	CFBP499	GCA_012922335.1
Xanthomonas hortorum pv. *vitians*	-	LM16734	GCA_014338485.1
Xanthomonas oryzae pv. *oryzae*	-	MAFF 311018	GCA_000010025.1
Xanthomonas perforans	-	CFBP 7293	GCA_001976075.1
Xanthomonas phaseoli pv. *dieffenbachiae*	-	LMG 25940	GCA_001401535.2
Xanthomonas phaseoli pv. *manihotis*	-	LMG784	GCA_001482665.1
Xanthomonas phaseoli pv. *phaseoli*	-	CFBP6984	GCA_900234425.1
Xanthomonas phaseoli pv. *phaseoli*	-	NCPPB 1420	GCA_000785935.2
Xanthomonas phaseoli pv. *phaseoli*	-	NCPPB 3035	GCA_000774035.2
Xanthomonas phaseoli pv. *syngonii*	-	LMG 9055	GCA_001640215.1
Xanthomonas sacchari	-	NCPPB 4393	GCA_000225975.2
Xanthomonas sp.	-	BRIP62411	GCA_003993525.1
Xanthomonas sp.	-	CPBF 426 2	GCA_903989465.1
Xanthomonas translucens	-	DAR61454	GCA_000334075.1
Xanthomonas translucens pv. *cerealis*	-	CFBP 2541	GCF_000807145.1
Xanthomonas translucens pv. *graminis*	-	ART-Xtg29	GCA_000313775.1
Xanthomonas translucens pv. *translucens*	-	DSM 18974	GCA_000331775.1
Xanthomonas translucens pv. *undulosa*	-	ICMP11055	GCA_003050685.1
Xanthomonas vasicola	-	NCPPB 989	GCA_000772795.1
Xanthomonas vasicola pv. *musacearum*	-	BCC282	GCA_003932615.1
Xanthomonas vasicola pv. *vasculorum*	-	SAM119	GCA_003015715.1
Xanthomonas vasicola pv. *zeae*	-	X01	GCA_003112105.1

Table S10a. Real-time *Ralstonia pseudosolanacearum* LAMP assay quencher & probe mix (Q-Mix) – Assay 1.[a]

Primer & Probe	Volume (µl)	Working Conc. (µM)
Q1-IBFQ (100 µM)	21	52.5
CP-LB-FAM (100 µM)	4	10
HP-LB-FAM (100 µM)	10	25
MG Water	5	N/A
**Total**	**40**	**N/A**

[a]The Q-mix is heated to 95°C for 5 min and then snap chilled on ice for 2 min.

Table S10b. Real-time *Ralstonia pseudosolanacearum* LAMP assay primers & probe mix (pMix) – Assay 1.

Primer & Probe	Volume (µl)	Working Conc. (µM)	Final Conc. (µM)
**Q-Mix[a]**	40	Q1-IBFQ: 2.1<br>CP-LB-FAM: 0.4<br>HP-LB-FAM: 1	Q1-IBFQ: 0.42<br>CP-LB-FAM: 0.08<br>HP-LB-FAM: 0.2
CP-F3 (100 µM)	10	1	0.2
CP-B3 (100 µM)	10	1	0.2
CP-FIP (100 µM)	80	8	1.6
CP-BIP (100 µM)	80	8	1.6
CP-LB (100 µM)	40	4	0.8
HP-F3 (100 µM)	10	1	0.2
HP-B3 (100 µM)	10	1	0.2
HP-FIP (100 µM)	80	8	1.6
HP-BIP (100 µM)	80	8	1.6
HP-LF (100 µM)	40	4	0.8
HP-LB (100 µM)	30	3	0.6
MG Water	490	N/A	N/A
**Total**	**1000**	**N/A**	**N/A**

[a]Q-mix should already be contained in the tube at a volume of 40 µl.

Table S11a. Real-time *Ralstonia pseudosolanacearum* phylotype I and III LAMP assay quencher & probe mix (Q-Mix) – Assay 2.[a]

Primer & Probe	Volume (µl)	Working Conc. (µM)
Q1-IBFQ (100 µM)	21	52.5
CP-LB-FAM (100 µM)	4	10
HP-LB-HEX (100 µM)	10	25
MG Water	5	N/A
**Total**	**40**	**N/A**

[a]The Q-mix is heated to 95°C for 5 min and then snap chilled on ice for 2 min.

Table S11b. Real-time *Ralstonia pseudosolanacearum* phylotype I and III LAMP assay primers & probe mix (pMix) – Assay 2.

Primer & Probe	Volume (µl)	Working Conc. (µM)	Final Conc. (µM)
**Q-Mix** [a]	40	Q1-IBFQ: 2.1<br>CP-LB-FAM: 0.4<br>HP-LB-HEX: 1	Q1-IBFQ: 0.42<br>CP-LB-FAM: 0.08<br>HP-LB-HEX: 0.2
CP-F3 (100 µM)	10	1	0.2
CP-B3 (100 µM)	10	1	0.2
CP-FIP (100 µM)	80	8	1.6
CP-BIP (100 µM)	80	8	1.6
CP-LB (100 µM)	40	4	0.8
HP-F3 (100 µM)	10	1	0.2
HP-B3 (100 µM)	10	1	0.2
HP-FIP (100 µM)	80	8	1.6
HP-BIP (100 µM)	80	8	1.6
HP-LF (100 µM)	40	4	0.8
HP-LB (100 µM)	30	3	0.6
MG Water	490	N/A	N/A
**Total**	**1000**	**N/A**	**N/A**

[a]Q-mix should already be contained in the tube at a volume of 40 µl.

Table S12a. Real-time *Ralstonia pseudosolanacearum* phylotype I LAMP assay quencher & probe mix (Q-Mix 1) – Assay 3.[a]

Primer & Probe	Volume (µl)	Working Conc. (µM)
Q1-IBFQ (100 µM)	8	20
CP-LB-FAM (100 µM)	4	10
MG Water	28	N/A
**Total**	**40**	**N/A**

[a]The Q-mix is heated to 95°C for 5 min and then snap chilled on ice for 2 min.

Table S12b. Real-time *Ralstonia pseudosolanacearum* phylotype III LAMP assay quencher & probe mix (Q-Mix 2) – Assay 3.[a]

Primer & Probe	Volume (µl)	Working Conc. (µM)
Q2-IBRQ (100 µM)	20	50
HP-LB-ATTO590 (100 µM)	10	25
MG Water	10	N/A
**Total**	**40**	**N/A**

[a]The Q-mix is heated to 95°C for 5 min and then snap chilled on ice for 2 min.

Table S12c. Real-time *Ralstonia pseudosolanacearum* phylotype I and III LAMP assay primers & probe mix (pMix) – Assay 3.

Primer & Probe	Volume (µl)	Working Conc. (µM)	Final Conc. (µM)
**Q-Mix 1[a]**	40	Q1-IBFQ: 0.8<br>CP-LB-FAM: 0.4	Q1-IBFQ:0.16<br>CP-LB-FAM: 0.08
Q-mix 2	40	Q2-IBRQ: 2.0<br>HP-LB-ATTO590: 1.0	Q2-IBRQ:0.4<br>HP-LB-ATTO590: 0.2
CP-F3 (100 µM)	10	1	0.2
CP-B3 (100 µM)	10	1	0.2
CP-FIP (100 µM)	80	8	1.6
CP-BIP (100 µM)	80	8	1.6
CP-LB (100 µM)	40	4	0.8
HP-F3 (100 µM)	10	1	0.2
HP-B3 (100 µM)	10	1	0.2
HP-FIP (100 µM)	80	8	1.6
HP-BIP (100 µM)	80	8	1.6
HP-LF (100 µM)	40	4	0.8
HP-LB (100 µM)	30	3	0.6
MG Water	450	N/A	N/A
**Total**	**1000**	**N/A**	**N/A**

[*]Q-mix should already be contained in the tube at a volume of 40 µl.

Table S13. Colorimetric *Ralstonia pseudosolanacearum* LAMP assay primer mix (pMix) – Assay 4.

Primer & Probe	Volume (µl)	Working Conc. (µM)	Final Conc. (µM)
CP-F3 (100 µM)	10	1	0.2
CP-B3 (100 µM)	10	1	0.2
CP-FIP (100 µM)	80	8	1.6
CP-BIP (100 µM)	80	8	1.6
CP-LB (100 µM)	40	4	0.8
HP-F3 (100 µM)	10	1	0.2
HP-B3 (100 µM)	10	1	0.2
HP-FIP (100 µM)	80	8	1.6
HP-BIP (100 µM)	80	8	1.6
HP-LF (100 µM)	40	4	0.8
HP-LB (100 µM)	40	4	0.8
MG Water	520	N/A	N/A
**Total**	**1000**	**N/A**	**N/A**

Table S14. Nucleotide sequence of the synthetic gBlocks™ positive controls for CP (conserved protein of unknown function; phylotype I-specific) and HP (hypothetical protein; phylotype III-specific) for detection and differentiation of *Ralstonia pseudosolanacearum* phylotypes I and III.

Rpseudo-I-CP
TGCCGCAAGGGGTGGCGCTCGCCGATGAGCAGCGGAAAGGCGTCGCGAATCTCCT<br>CGGCAATCTGCAGCACCAGCGGTGCGTTGAAGCCGGTGAAGAACAGCGTGCTCAA<br>CCGGCCGTGGGCATAGCGGTTGCCGGCCCAGATCGTGGAGTCCTGGCAGAAGCGG<br>AGCAGGCCCCGGAGGGCCTCGTCCGTCAGGAAATCGTCGATGACCACCACGTTGG<br>GGCGGTGTTCCAGGTAGCGGCGCTGGACCT
Rpseudo-III-HP
GGCCAGCCTGGCGTGCCCGTCACCGCAGGCGTGGCGCTGCTGGCGAACATGATGG<br>CCGATCGCTACCTGGATCGGGGTGAAGCCGCCGACCAGGCCGAGTGGGTGAACCC<br>GCTCAAGGGTTTGCCGATGGTGGCGGGTATCAAGCACATGCTGAGCCGGCAACAG<br>CGACGCACGGAATGGACATGCGTGCGCCCGCCGCTGCAGGTGCTGAGCAGTACGC<br>AACGACAACGGCTGGACCGCTGGATGTCAC
